# The interplay between tissue‐resident microbiome and host proteins by integrated multi‐omics during progression of colorectal adenoma to carcinoma

**DOI:** 10.1002/imt2.70090

**Published:** 2025-11-04

**Authors:** Di Wu, An‐Jun Wang, De‐Chao Bu, Yan‐Yan Sun, Chen‐Hao Li, Yue‐Mei Hong, Shan Zhang, Shi‐Yang Chen, Jin‐An Zhou, Tian‐Yi Zhang, Min‐Hao Yu, Yong‐Jing Ma, Xiu‐Li Wang, Jia Xu, Wei He, Christopher Heeschen, Jian‐Feng Chen, Wen‐Jun Mao, Hui Ding, Wen‐Juan Wu, Yi Zhao, Hui Wang, Ning‐Ning Liu

**Affiliations:** ^1^ State Key Laboratory of Systems Medicine for Cancer, Center for Single‐Cell Omics, School of Public Health Shanghai Jiao Tong University School of Medicine Shanghai China; ^2^ Shanghai Xuhui Center for Disease Prevention and Control (Shanghai Xuhui Health Inspection Agency) Shanghai China; ^3^ Research Center for Ubiquitous Computing Systems, Institute of Computing Technology Chinese Academy of Sciences Beijing China; ^4^ University of Chinese Academy of Sciences Beijing China; ^5^ Key Laboratory of Systems Health Science of Zhejiang Province, School of Life Science, Hangzhou Institute for Advanced Study University of Chinese Academy of Sciences Hangzhou China; ^6^ Department of Gastrointestinal Surgery, Renji Hospital Shanghai Jiao Tong University School of Medicine Shanghai China; ^7^ Department of Pathology, Renji Hospital Shanghai Jiao Tong University School of Medicine Shanghai China; ^8^ Department of Immunology, School of Basic Medical Sciences Fudan University Shanghai China; ^9^ Department of Thoracic Surgery The Affiliated Wuxi People's Hospital of Nanjing Medical University, Wuxi People's Hospital, Wuxi Medical Center, Nanjing Medical University Wuxi China; ^10^ Center of Clinical Research The Affiliated Wuxi People's Hospital of Nanjing Medical University, Wuxi People's Hospital, Wuxi Medical Center, Nanjing Medical University Wuxi China; ^11^ Department of Gastroenterology and Hepatology, Renji Hospital Shanghai Jiao Tong University School of Medicine, Shanghai Jiao Tong University Shanghai China; ^12^ Shanghai Institute of Digestive Disease NHC Key Laboratory of Digestive Diseases Shanghai China; ^13^ Department of Laboratory Medicine, Shanghai East Hospital Tongji University School of Medicine Shanghai China; ^14^ Hainan International Medical Center Shanghai Jiao Tong University School of Medicine, Haikou Hainan China

**Keywords:** colorectal cancer, metagenomics, microbiota‐host interaction, proteomics, tissue‐resident microbiome

## Abstract

The intratumoral microbiome is an emerging hallmark of cancer, yet its multi‐kingdom host–microbiome ecosystem in colorectal cancer (CRC) remains poorly characterized. Here, we conducted an integrated analysis using deep shotgun metagenomics and proteomics on 185 tissue samples, including adenoma (A), paired tumor (T), and para‐tumor (P). We identified 4057 bacterial, 61 fungal, 108 archaeal, and 374 viral species in tissues and revealed distinct intratumor microbiota dysbiosis, indicating a CRC‐specific multi‐kingdom microbial ecosystem. Proteomic profiling uncovered four CRC subtypes (C1–C4), each with unique clinical prognoses and molecular signatures. We further discovered that host‐microbiome interactions are dynamically reorganized during carcinogenesis, where different microbial taxa converge on common host pathways through distinct proteins. Leveraging this interplay, we identified 14 multi‐kingdom microbial and 8 protein markers that strongly distinguished A from T samples (area under the receiver operating characteristic curve (AUROC) = 0.962), with external validation in two independent datasets (AUROC = 0.920 and 0.735). Moreover, we constructed an early‐ versus advanced‐stage classifier using 8 microbial and 4 protein markers, which demonstrated high diagnostic accuracy (AUROC = 0.926) and was validated externally (AUROC = 0.659–0.744). Functional validation in patient‐derived organoids and murine allograft models confirmed that enterotoxigenic *Bacteroides fragilis* and *Fusobacterium nucleatum* promoted tumor growth by activating Wnt/β‐catenin and NF‐κB signaling pathways, corroborating the functional potential of these biomarkers. Together, these findings reveal dynamic host–microbiome interactions at the protein level, tracing the transition from adenoma to carcinoma and offering potential diagnostic and therapeutic targets for CRC.

## Highlights

- Integrated metagenomic and proteomic profiling of 185 colorectal tissue samples. and identification of colorectal cancer (CRC)‐specific multi‐kingdom microbial ecosystems and four molecular subtypes with distinct clinical features.
- Discovery of 14 microbial and 8 protein markers with high diagnostic accuracy for adenoma versus tumor and early versus advanced stage classification.
- Functional validation of *Bacteroides fragilis* and *Fusobacterium nucleatum* as key microbial drivers promoting tumor growth via Wnt/β‐catenin and NF‐κB signaling.
- Insights into dynamic host–microbiome interactions with potential diagnostic and therapeutic relevance.

## Graphical abstract

This study integrated metagenomic and proteomic profiling of 185 colorectal tissue samples—spanning adenoma (A), tumor (T), and para‐tumor (P)—to characterize multi‐kingdom microbiome and host protein dynamics in colorectal cancer (CRC). In total, 4057 bacterial, 61 fungal, 108 archaeal, and 374 viral species were identified, revealing CRC‐specific microbial dysbiosis. Proteomic analysis defined four molecular subtypes (C1–C4) with distinct clinical outcomes. This study further developed a diagnostic model based on 14 microbial and 8 protein markers, which robustly distinguished adenoma from tumor (achieved an area under the receiver operating characteristic curve (AUROC) = 0.962) and early from advanced stages (AUROC = 0.926), with validation across multiple independent cohorts. Functional assays in patient‐derived organoids and murine allografts confirmed that enterotoxigenic *Bacteroides fragilis* and *Fusobacterium nucleatum* promote tumor growth through Wnt/β‐catenin and NF‐κB pathway activation. These findings highlight dynamic host–microbiome interactions in CRC progression and provide novel biomarkers and therapeutic targets.

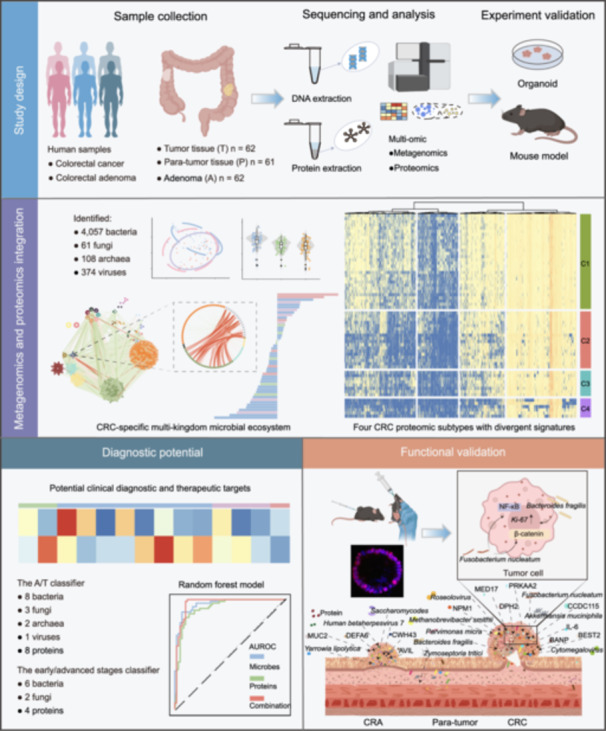

## INTRODUCTION

Colorectal cancer (CRC) is the third most common cancer worldwide, accounting for ~10% of all cancer diagnoses and ranking as the second leading cause of cancer‐related mortality [1, 2]. Alarmingly, CRC incidence is rising, particularly in low‐income countries and among younger populations [3, 4]. Colorectal adenomas (CRA) are well‐established precursors of most CRC cases [1, 5]. Over time, some adenomas progressively accumulate mutations, advancing to high‐grade adenomas or invasive carcinoma [4, 6]. Early diagnosis of CRC at the precancerous adenoma stage is critical, as it markedly improves the 5‐year survival rate to ~90% and significantly reduces both incidence and mortality, thereby alleviating the global health burden [1, 7]. Timely detection and removal of adenomas are thus essential to interrupt the adenoma–carcinoma sequence. However, predicting which adenomas will undergo malignant transformation remains *a major* challenge, as not all adenomas progress to CRC.

Environmental factors—including obesity, alcohol consumption, and smoking—are established CRC risk factors [8, 9]. More recently, polymorphic microbiomes have been recognized as a hallmark of cancer [10]. Microbial dysbiosis contributes to colorectal carcinogenesis through diverse mechanisms, including the production of toxic metabolites [11], induction of intestinal inflammation [12], and modulation of host immune responses [13]. Intratumoral bacteriomes have been documented across multiple solid tumors and may facilitate metastatic progression [14, 15]. For instance, increased abundance of *Streptococcus* correlates with upregulation of host genes involved in Wnt and NF‐κB signaling pathways [16, 17]. Moreover, pan‐cancer studies have revealed cancer‐type‐specific fungal ecologies with diagnostic and prognostic potential [18, 19]. Notably, CRC patients display strengthened fungal–bacterial associations within the gut microbiome [20].

Advances in multi‐omics technologies have further illuminated the molecular and microbial heterogeneity of CRC and their links to diverse clinical outcomes [21, 22]. Both protein and microbial biomarkers show promise for improving CRC diagnosis and may enable cost‐effective screening [7, 20, 23, 24, 25, 26]. Although intestinal tissue has been extensively studied as an interface for host–microbiota interactions during tumorigenesis [27, 28], the complexity of microbiota–host interactions within the heterogeneous tumor microenvironment (TME) in CRC remains incompletely understood.

In this study, we performed integrated multi‐omic analyses to investigate interactions between tissue‐resident multi‐kingdom microbiota and host proteins throughout the adenoma–carcinoma transition. Functional validation using patient‐derived organoids and murine allograft models demonstrated that enterotoxigenic *Bacteroides fragilis* (ETBF) and *Fusobacterium nucleatum* promote colorectal carcinogenesis by activating Wnt/β‐catenin and NF‐κB signaling pathways. We further evaluated the diagnostic performance of combined microbial and protein biomarkers in differentiating CRA from carcinomas, as well as early‐ from advanced‐stage CRC. Our microbiota–host‐centric analytical strategy provides a framework to deepen understanding of microbiota–host interplay in CRC progression and to guide future biomarker and therapeutic development.

## RESULTS

### Multi‐omic design of a colorectal tissue cohort for host‐microbiome integration

We performed a comprehensive multi‐omic analysis of 185 tissue samples obtained from 124 patients at Renji Hospital in Shanghai, encompassing both CRA and colorectal cancer cases. The study design included in‐depth analysis of adenoma tissues (A), paired tumor (T), and para‐tumor (P) tissues, together with phenotypic data. The three groups were comparable in terms of age, sex, and body mass index (BMI) (*p* > 0.05) (Table S1). Among the CRC patients, 33 (53.23%) were early stage (Stage I/II) and 29 (46.77%) were advanced stage (Stage III/IV). The cohort comprised 19 proximal and 43 distal tumors. Rigorous inclusion and exclusion criteria were applied to ensure cohort homogeneity and selection accuracy.

**Figure 1:**
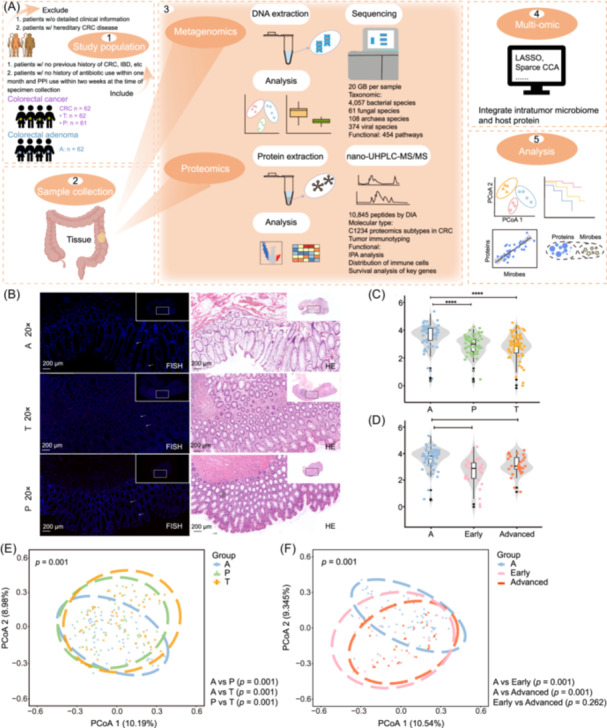
Schematic overview of this study and the tissue‐resident microbiome analysis. (A) The cohort assessed tissue‐related microbiota of 62 colorectal cancer (CRC) and colorectal adenoma (CRA) patients. Phenotypic data through in‐person assessment including: (1) personal characteristics (age, sex, body mass index (BMI)). (2) lifestyle factors (physical activity, alcohol consumption, smoking, food frequency) were assessed by international standard questionnaire. Multi‐omics including metagenomics and proteomics were applied in the cohort. Data analysis and validation were carried out from microbiome and host, respectively, and tissue‐related microbiome was compared with intestinal microbiome and oral microbiome. (B) Representative hematoxylin and eosin (HE) staining and fluorescence in situ hybridization (FISH) using probes against fungal 28S rRNA of adenoma (A), para‐tumor (P: >5 cm to cancer focus) and tumor (T). Scale bars, 200 µm. (C) Overall microbiome alpha diversity measured by Shannon index of A (blue, *n* = 62), P (green, *n* = 61), and T (yellow, *n* = 62). The *p*‐value (Kruskal–Wallis test) was calculated. Data are shown via the interquartile ranges (IQRs) with the median as a black horizontal line and the whiskers extending up to the most extreme points within 1.5× the IQR; outliers are represented as dots. NS, not significant. (D) Overall microbiome alpha diversity measured by Shannon index of A (blue, *n* = 62), early (pink, *n* = 32), and advanced (red, *n* = 29). The *p*‐value (Kruskal–Wallis test) was calculated. Data are shown via the interquartile ranges (IQRs) with the median as a black horizontal line and the whiskers extending up to the most extreme points within 1.5× the IQR; outliers are represented as dots. NS, not significant. (E) Principal coordinate analysis (PCoA) of samples based on Bray–Curtis distance, which shows that microbial composition was different between A (blue), P (green), and T (yellow). The *p* values of beta diversity based on Bray–Curtis distance were calculated with PERMANOVA by 999 permutations (two‐sided test). (F) Principal coordinate analysis (PCoA) of samples based on Bray–Curtis distance, which shows that microbial composition was different between and A (blue), early (pink), and advanced (red). The *p* values of beta diversity based on Bray–Curtis distance were calculated with PERMANOVA by 999 permutations (two‐sided test). DIA, data independent acquisition; IBD, inflammatory bowel diseasey; IPA, ingenuity pathway analysis; LASSO, least absolute shrinkage and selection operator; nano‐UHPLC‐MS/MS, nano‐ultra high performance liquid chromatography‐tandem mass spectrometr; PPI, proton pump inhibitors; Sparce CCA, sparse canonical correlation analysis.

To enable broad‐spectrum microbial profiling, we employed fungi‐enriched DNA extraction and deep shotgun metagenomic sequencing at a depth of ~20 Gb per sample, identifying 4057 bacterial, 61 fungal, 108 archaeal, and 374 viral species. In parallel, proteomic profiling was conducted using data‐independent acquisition (DIA), enabling an integrated host–microbe analysis (Figure 1A, Figure S1A–C).

### Tissue‐resident microbiome dysbiosis in CRC patients

Low‐biomass microbiome studies are inherently susceptible to contamination, which can compromise the validity of the results. To address this, we implemented rigorous environmental controls, including strict hospital and laboratory procedures, optimized DNA extraction protocols, and comprehensive negative controls (Figure S1D,E). We analyzed 185 tissue samples together with 6 control samples and applied the Decontam package [29], which removed 3282 potential contaminants (Figure S1D–F and Table S2–S4).

We first used fluorescence in situ hybridization (FISH) to confirm the presence of tissue‐resident mycobiota within tumor tissues (Figure 1B). This was further validated by polymerase chain reaction (PCR), where amplification with both 16S and ITS2 primers confirmed the presence of bacterial and fungal DNA in tissue samples but not in controls (Figure S1E). Mycobiome enrichment was notably higher in T compared with A or P.

We next assessed the overall tissue‐resident multi‐kingdom microbiome. Alpha diversity was significantly reduced in T compared with A (*p* < 0.0001), a pattern consistent with previous gut microbiome studies [20, 30, 31, 32] (Figure 1C). In total, 3616, 4155, and 4225 microbial species were identified in A, P, and T, respectively (Tables S5–S10). Interestingly, no significant differences were observed between early‐ and advanced‐stage tumor tissues, in agreement with prior reports [33] (Figure 1D). Principal coordinate analysis based on Bray–Curtis matrices further revealed distinct beta diversity of microbial communities at the species level among A, P, and T samples (all between‐group *p* = 0.001; Figure 1E). Significant compositional shifts were also evident between A and both early‐ and advanced‐stage tumors. In contrast, no statistically significant difference was observed between early‐ and advanced‐stage tumors (*p* = 0.262; Figure 1F).

Together, these data highlight intratumoral microbiome dysbiosis in CRC compared with adenomas, while indicating that microbial community structures are broadly conserved between early‐ and advanced‐stage tumors.

### Alterations in the multi‐kingdom microbiome during cancer progression

**Figure 2:**
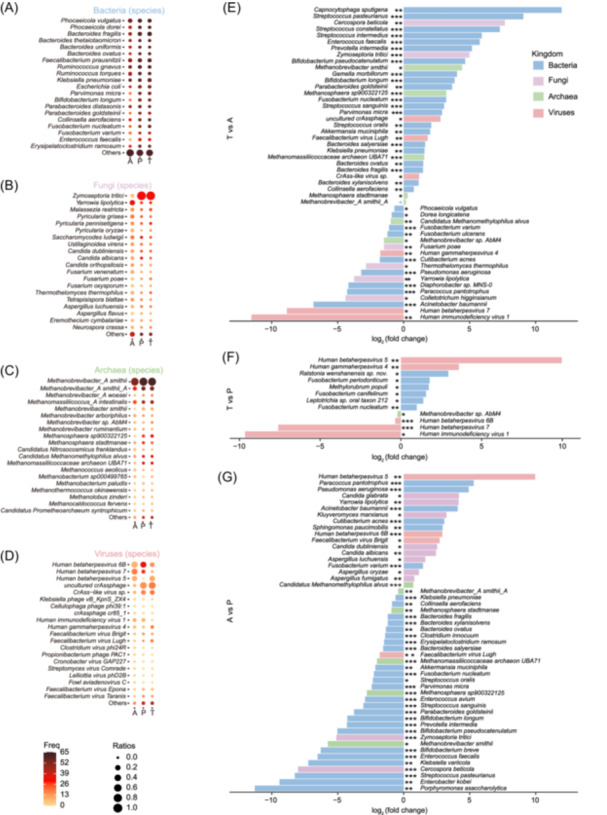
Landscape of multi‐kingdom tissue‐resident microbial composition from adenoma to cancer. (A) Taxonomic composition of bacteria in A, P, and T at the species level. Color strength indicates the recurrence of microbes in the sample, and ratios represent the percentage of microbes. Only the abundant species are shown in the bubble chart, and the rare families are summed into others. (B) Taxonomic composition of fungi in A, P, and T at the species level. Visualization and annotation as in (A). (C) Taxonomic composition of archaea in A, P, and T at the species level. Visualization and annotation as in (A). (D) Taxonomic composition of viruses in A, P, and T at the species level. Visualization and annotation as in (A). (E) Differentially abundant microbes in T versus A. Vertical coordinates indicate microbial names, and the horizontal coordinates indicate the fold change in microbial abundance after taking log_2_. (F) Differentially abundant microbes in T versus P. Axes and annotation as in (E). (G) Differentially abundant microbes in A versus P. Axes and annotation as in (E). The different colors represent the different microorganisms in the four kingdoms. *p < 0.05, **p < 0.01, ***p < 0.001. Freq, frequency.

We next characterized microbial composition across tissue types at multiple taxonomic levels. The bacterial and fungal profiles were more closely aligned in T and P tissues at both the genus and species levels, whereas archaeal composition remained relatively consistent across A, P, and T samples (Figure 2A–D, Figure S2A).

Within A samples, we observed a substantial reduction in *Saccharomycodes*, accompanied by marked increases in *Bacteroides* and *Fusobacterium* species, including *Fusobacterium nucleatum*, *Bacteroides fragilis*, *Parvimonas micra*, and *Prevotella intermedia*. These shifts are consistent with previous studies [1, 20, 34, 35, 36, 37, 38, 39]. Although the microbiome was largely dominated by genera such as *Bacteroides*, *Phocaeicola*, *Faecalibacterium*, *Streptococcus*, and *Mediterraneibacter*, tumor tissues exhibited elevated abundances of *Bacteroides* and *Klebsiella*, consistent with gut microbiome profiles reported in CRC patients [1, 40]. (Figure 2E–G and Tables S11–S14).

Compared with P tissues, T tissue samples showed a higher abundance of *F. nucleatum*, while *F. nucleatum*, *B. fragilis*, *P. micra*, and *Porphyromonas asaccharolytica* were significantly reduced in A samples. Microbial composition between early‐ and advanced‐stage tumors appeared broadly similar, with the exception of viral communities, suggesting a possible role for viruses in CRC progression that warrants further investigation (Figure S2B,C).

Collectively, these findings highlight distinct intratumoral microbiota dysbiosis, characterized by enrichment of carcinogenic microbial biomarkers in T relative to A.

### Microbial community stratification reveals adenoma‐to‐carcinoma ecological shifts

To further explore ecological dynamics, we applied community typing (clustering) using the Dirichlet multinomial mixtures (DMM) model at the genus level [41]. This analysis identified four distinct microbial clusters (M1–M4) (Table S15). M1 was represented by major bacterial genera and a fungal family, including *Streptococcaceae*, *Streptococcus*, *Moraxellaceae*, *Acinetobacter*, and *Yarrowia*, while M2 was largely characterized by *Bacteroidaceae*, *Bacteroides*, and *Phocaeicola*. M3 consisted mainly of *Oscillospiraceae*, *Lachnospiraceae*, and *Faecalibacterium*. M4 was enriched in *Enterobacteriaceae*, *Escherichia*, and *Klebsiella*, including *Escherichia coli* and *Klebsiella pneumoniae*.

**Figure 3:**
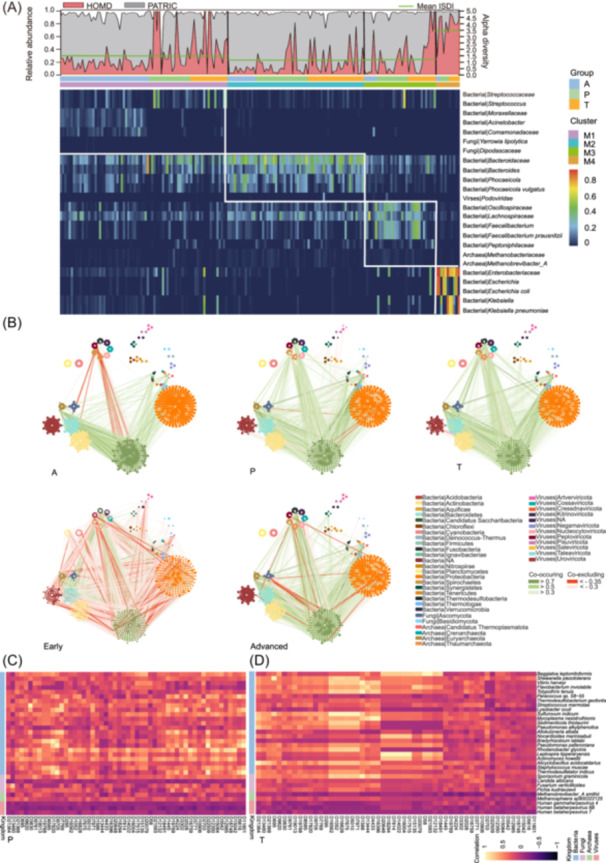
Co‐abundance correlations and functional prediction among multi‐kingdom species in colorectal carcinogenesis. (A) The metacommunities are defined by fitting microbiome data to the Dirichlet multinomial mixtures (DMM) model. Reads for oral strains or known pathogenic strains considered likely to originate in the human gut were classified according to a collection of 16S rRNA genes from the Human Oral Microbiome (HOM; version 13) database (https://homd.org/taxa/tax_table) and the PATRIC bacterial pathogens database(https://www.bv-brc.org/). (B) The interaction networks of all four kingdoms between each group at the genus level. Estimation of correlation using SparCC algorithm. The colors of nodes indicate genus, and the size of nodes indicates the abundance of genus. A hollow indicates that the microbe is not present in the group. Only significant absolute correlations above 0.3 are shown, which are considered as fair correlations. The red lines indicate negative interactions; the green lines indicate positive interactions. (C) The heatmap of Spearman correlations between differential microbes and metabolic pathways in P. Displayed are the top 50 most differential bacterial and top 10 fungal/archaeal/viral species‐pathway pairs, ranked by the absolute difference in correlation coefficients between groups. (D) The heatmap of Spearman correlations between differential microbes and metabolic pathways in T. Displayed are the top 50 most differential bacterial and top 10 fungal/archaeal/viral species‐pathway pairs, ranked by absolute difference in correlation coefficients between groups. ISDI, inverse Simpson diversity index.

Among these, M4 exhibited the lowest diversity, as reflected by the inverse Simpson index. Notably, M4 contained a higher abundance of oral taxa, with nearly identical 16S rRNA reads to those from the Human Oral Microbiome and PATRIC bacterial pathogen databases (Figure 3A). Cluster distribution also reflected disease progression: 66.13% of adenomas were classified as M1, with the proportion of A samples progressively decreasing from M1 to M4. In contrast, M4 was predominantly composed of T and P samples, underscoring a shift in microbial community structure from adenoma to carcinoma.

### Multi‐kingdom microbial interaction networks reveal ecological reorganization during CRC progression

Recognizing the importance of multi‐kingdom microbial interactions within the tumor micro‐ecosystem during CRC progression [20], we conducted sparse correlations for compositional data (SparCC) analysis [42] on metagenomic sequencing data from A, P, and T samples, including both early‐ and advanced‐stage tumor tissues.

At the genus level, both the number and strength of correlations, whether positive or negative, were minimal in adenomas (Figure 3B). In contrast, at the species level, negative correlations became more prominent in early‐ and advanced‐stage tumor tissues (Figure S3A), indicating distinct ecological niches across groups. This pattern was likely driven by the enrichment of pathogenic bacteria within the TME, such as *F. nucleatum* and *B. fragilis* (Figure 2A–D). To highlight key associations, we focused our interaction analyses on Ascomycota. Compared with A and P, the negative correlations in T between Ascomycota and other taxa (especially Firmicutes) increased and intensified with disease severity, peaking in early CRC, and subsequently shifted toward positive correlations with Proteobacteria in the tumor stage (Figure S3B).

Given the substantial variability among individual microbiota, it is plausible that different microbes across individuals may fulfill similar functions through shared pathways. To explore these functional relationships between microbiota and host, we performed Spearman correlation analyses of microbial species and pathways in P and T tissues. Strikingly, contrasting correlation patterns emerged (Figure 3C,D, Figure S3C, and Tables S16–S19). For example, the transposase IS116/IS110/IS902 family, peptidase M16, and the major facilitator superfamily were negatively associated with 12.5%, 30%, and 15.63% of microorganisms in P, respectively, but these proportions shifted to 9.38%, 9.38%, and 12.5% in T.

Importantly, compared with bacterial microbiota, which exhibited predominantly positive correlations with pathways, microbiota from other kingdoms displayed more negative associations. Specifically, *Candida albicans* showed nearly universal negative correlations with pathways in P, but this trend was partially reversed in T, suggesting a group‐dependent functional role.

Collectively, these results reveal profound alterations in tissue‐resident microbial interactions and functions across A, P, and T, underscoring the dynamic reorganization of the multi‐kingdom microbiome during CRC progression.

### Proteomic profiling identifies four CRC subtypes with distinct immune evasion phenotypes

We next examined the intricate interactions between tissue‐resident microbiota and host proteins during CRC progression. Although it is well established that microbiota influence colorectal tumorigenesis through metabolites and proteins [43, 44], protein‐level interactions throughout CRC development remain underexplored. To address this gap, we conducted deep proteomic profiling of both adenoma and tumor tissues using nano‐ultra high performance liquid chromatography‐tandem mass spectrometry (nano‐UHPLC‐MS/MS), characterized via DIA‐based methods, identifying 10,845 distinct proteins (Table S20).

**Figure 4:**
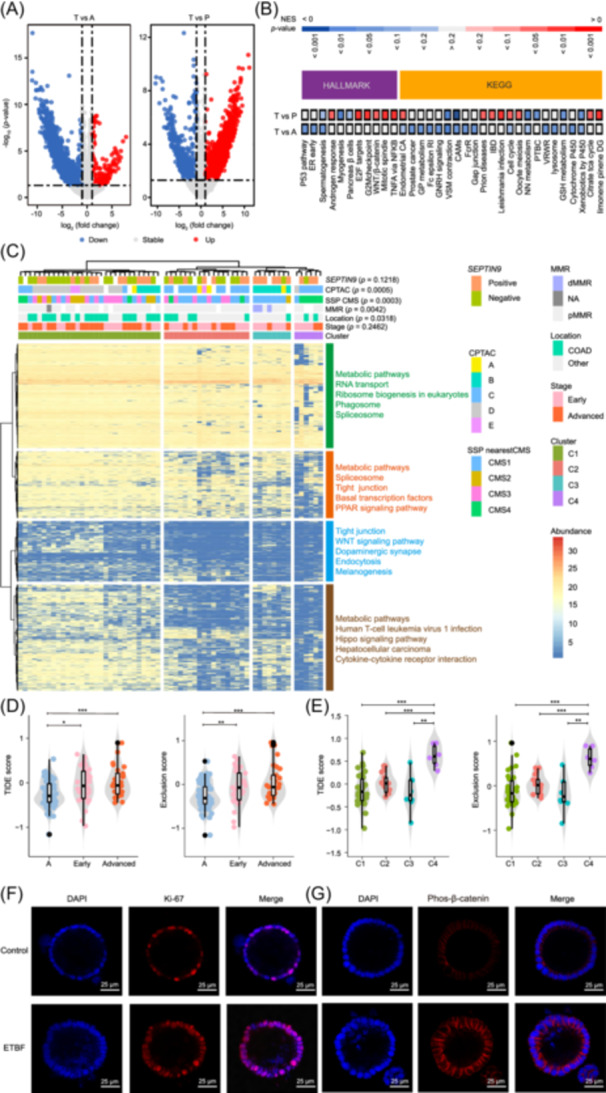
Proteomic subtyping of adenoma, para‐tumor, and tumor tissues based on differentially expressed proteins. (A) Differential expressed proteins in T vs A and T versus P. Volcano plot illustrates the overlap of differential proteins in T versus A and T versus P. Red dots are upregulated and blue dots are downregulated. Paired *t*‐test was used for differences between T versus P, and two‐sample *t*‐test was used for differences between T versus A. Two‐tailed Student's *t*‐test was used for significance test and Benjamini–Hochberg (BH) correction. (B) Gene set enrichment analysis of T versus P and T versus A. The red bars represent gene sets with upregulated genes and the blue bars represent gene sets with downregulated genes. (C) Consensus clustering based on differentially expressed proteins. All subjects were divided into four subgroups, named C1–C4. The bars above the heatmap represent SEPTIN9, CPTAC, CMS, MMR, Location, Stage, and Cluster, respectively. Each column represents a patient sample and rows indicate proteins. (D) Tumor immune dysfunction and exclusion (TIDE) score in each group. Different colors represent different groups. Data are shown via the interquartile ranges (IQRs) with the median as a black horizontal line and the whiskers extending up to the most extreme points within 1.5 × the IQR; outliers are represented as dots. *p* value (two‐sided test) was calculated. NS, not significant. (E) Tumor immune dysfunction and exclusion score in molecular subtypes of proteomics. Different colors represent different groups. Data are shown via the interquartile ranges (IQRs) with the median as a black horizontal line and the whiskers extending up to the most extreme points within 1.5 × the IQR; outliers are represented as dots. *p* value (two‐sided test) was calculated. (F) Representative images of organoids and Enterotoxigenic *Bacteroides fragilis* (ETBF) after 4 h of Coculture with fluorescently labeled organoids showing increased proliferative activity (DAPI, blue, left; cell proliferation dye Ki‐67, red, middle; merged on the right panel). (G) Representative images of organoids and ETBF after 4 h of coculture with fluorescently labeled organoids showing activation of Wnt signaling (DAPI, blue, left; phosphorylation of β‐catenin changes dye, red, middle; merged on the right panel). Scale bar, 25 μm. CMS, consensus molecular subtype; COAD, colon adenocarcinoma; CPTAC, clinical proteomic tumor analysis consortium; KEGG, kyoto encyclopedia of genes and genomes; MMR, mismatch repair; NES, normalized enrichment scores.

Although para‐tumor tissues may not represent perfect normal controls, they are widely used in tumor studies [14, 45, 46]. Our analysis revealed clear differences in protein expression patterns between A and P. (Tables S21–S23) When comparing T with P, 2338 proteins were significantly upregulated, while 509 showed a notable decrease. Similarly, comparison of T versus A identified 3453 differentially expressed proteins (DEPs), including 184 upregulated and 3269 downregulated proteins (Figure 4A).

Pathway enrichment analysis using Kyoto Encyclopedia of Genes and Genomes (KEGG) and hallmark gene sets revealed cancer‐associated pathways significantly altered in T versus P (Figure 4B). Tumor tissues showed enrichment of proliferation‐associated pathways (E2F Targets, G2M Checkpoint, and Mitotic Spindle), along with oncogenic signaling including Wnt/β‐catenin and NF‐κB. In contrast, pathways such as p53 signaling, a canonical tumor suppressor pathway, were significantly downregulated in T versus A [47], alongside broad suppression of metabolic pathways.

To further explore molecular heterogeneity among tumor subtypes, we conducted hierarchical clustering of 2,847 DEPs between P and T, identifying four distinct proteomic clusters (C1–C4) (Figure 4C, Figure S4A). C1 was characterized by enrichment of metabolic and RNA transport pathways. C2 featured upregulation of proteins involved in tight junctions, basal transcription factors, and PPAR signaling. C3 was marked by downregulation of tight junction and Wnt signaling pathways, while C4 was defined by reduced activity in metabolic and Hippo pathways (Figure 4C). Moreover, hierarchical clustering of A and T also revealed partial overlap, with adenomas sharing some molecular features with tumors despite distinct clinical characteristics (Figure S4B).

When mapped to established consensus molecular subtypes (CMS) [48] and clinical proteomic tumor analysis consortium (CPTAC) subtypes [49], our clusters aligned well with previous classifications. Specifically, C3 and C4 corresponded with CMS1 and CMS4, as well as CPTAC subtypes B and C. The C3 subtype, associated with microsatellite instability (CMS1/CPTAC‐B), suggests potential suitability for immunotherapy. Conversely, the C4 subtype—linked to CMS4/CPTAC‐C and typically associated with poor prognosis—was more frequent in colon cancers, whereas rectal cancers were more often classified as C2, C3, or C4. Notably, tumor immune dysfunction and exclusion (TIDE) scores were significantly higher in T compared with A, indicating possible tumor immune escape within the TME (Figure 4D). Among the subtypes, C4 exhibited the highest TIDE score, suggesting a profoundly immunosuppressive microenvironment and reduced likelihood of responding to immune checkpoint blockade (Figure 4E).

To functionally validate microbiota‐driven pathway activation, we cocultured enterotoxigenic *Bacteroides fragilis* (ETBF) with patient‐derived colorectal cancer organoids. ETBF infection (MOI = 50) significantly increased expression of the proliferation marker Ki67 and phosphorylated β‐catenin (Figure 4F,G), confirming activation of Wnt/β‐catenin signaling and epithelial proliferation. These findings align with previous reports that ETBF's BFT toxin directly modulates β‐catenin stability and promotes colorectal carcinogenesis [50, 51, 52], further supporting our proteomic observations.

Together, these results highlight global rewiring of host proteins and pathways during CRC progression, define four proteomic subtypes with distinct clinical implications, and provide functional evidence for microbiota‐driven activation of oncogenic signaling.

### The regulatory network of protein–protein interactions in tumor tissues

**Figure 5:**
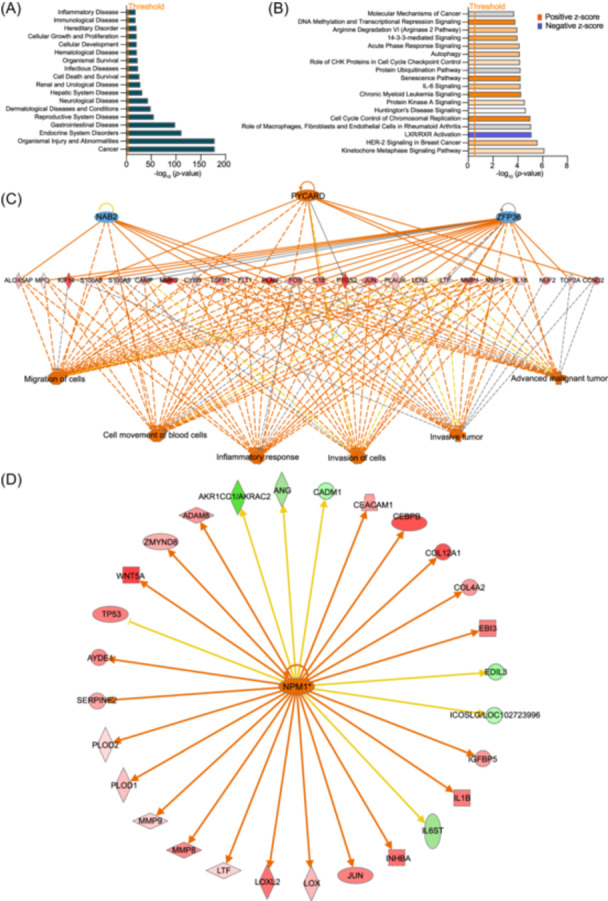
**E**xploration of protein–protein interactions and protein networks relevant in para‐tumor and tumor tissues by IPA analysis. (A) Disease and functional pathways annotated with differentially expressed proteins. Threshold denotes −log (*p*‐value). (B) Classical pathways enriched with differentially expressed proteins, Threshold denotes −log (*p*‐value), orange denotes positive *z*‐score, i.e., *z*‐score > 2, blue denotes negative a‐score, i.e., *z*‐score < −2. score < −2. Shades of color indicate the extent to which the pathway is activated or repressed; gray indicates that the activation and repression of the pathway cannot be calculated, and white indicates that it is not possible to indicate whether the pathway is activated or repressed. The significance test was performed using Fisher's exact probability method on the right. |*z*‐score | > 2, and *p* < 0.05 was statistically significant. (C) Gene interaction network diagram showing the molecular interactions in the network with the highest score under the conditions of this experiment. The different molecule types are still labeled with different shapes in the network diagram; the fill color and shade of the molecule indicate the fold change size and orientation of the molecule in the experimental results; the lines, arrows, and labels annotate the molecular interactions. (D) Upstream regulatory network diagram showing the interactions between upstream regulators and their directly related downstream molecules present in the data set. Orange lines indicate consistent activation of expression status between upstream regulators and genes, blue lines indicate consistent repression of expression status between upstream regulators and genes, while yellow lines indicate inconsistent expression status between upstream regulators and genes, and gray lines indicate the absence of predictive information related to expression status in the data set. Significance tests were performed using Fisher's exact probability method on the right. |z‐score| > 2 and *p* < 0.05 are statistically significant.

To better understand the complexity of intratumoral protein–protein interactions, we utilized ingenuity pathway analysis (IPA). Compared with P tissues, T samples exhibited significant enrichment in disease‐ and function‐related categories closely associated with CRC, particularly cancer and gastrointestinal diseases (Figure 5A). A key finding was the notable inhibition of the LXR/RXR activation pathway in T (Figure 5B). Since LXR/RXR are critical regulators of cholesterol and fatty acid homeostasis [53], this inhibition suggests potential dysregulation of cholesterol metabolism within tumors.

In contrast, several pathways were significantly activated in T, including the senescence pathway, IL‐6 signaling, and 14‐3‐3–mediated signaling. Tumor tissues also exhibited marked activation of processes related to cell migration, invasion, and immune responses, with particularly strong activation observed in aggressive tumor subtypes (Figure 5C).

Upstream regulatory network analysis further highlighted key drivers of these processes (Figure 5D). *NPM1* emerged as an upstream regulator and was found to activate a range of downstream genes, including *CEBPB*. Notably, *NPM1* has been reported to upregulate *PD‐L1* transcription, thereby inhibiting T‐cell activity and promoting tumor growth in triple‐negative breast carcinoma [54]. Meanwhile, *CEBPB*, a well‐established regulator of inflammatory responses [55] and tumorigenesis [56], may functionally interact with *NPM1*, suggesting a synergistic role in CRC progression.

Together, these results reveal an intricate network of protein–protein interactions and regulatory pathways, underscoring the complexity of molecular crosstalk within the TME and highlighting host proteins as potential therapeutic targets in CRC.

### Shared and distinct signatures of stage‐specific host–microbiome interactions in A, P, and T

The interaction between host and microbiome plays a crucial role in CRC development and progression [57, 58]. Despite extensive efforts to characterize CRC proteomic profiles, the dynamic interplay between the microbiome and host proteins remains less well explored. In our study, CRC samples were divided into four molecular subtypes (C1–C4) based on proteomic profiling. Notably, alpha diversity differed significantly among subtypes, with C4 showing the highest alpha diversity, suggesting a potential link between poor prognosis in C4 and intratumoral microbiota dysbiosis. However, no significant differences in beta diversity were observed (Figure S5A,B). We further correlated tumor‐enriched microbiota with TIDE scores. Tumor tissue‐enriched microbiota in advanced‐stage CRC and the C4 subtype were largely positively correlated with TIDE scores, implicating a potential role in facilitating immune evasion during CRC progression (Figure S5C). In particular, a significant positive correlation between *P. intermedia* and TIDE was observed in the C4 subtype.

**Figure 6:**
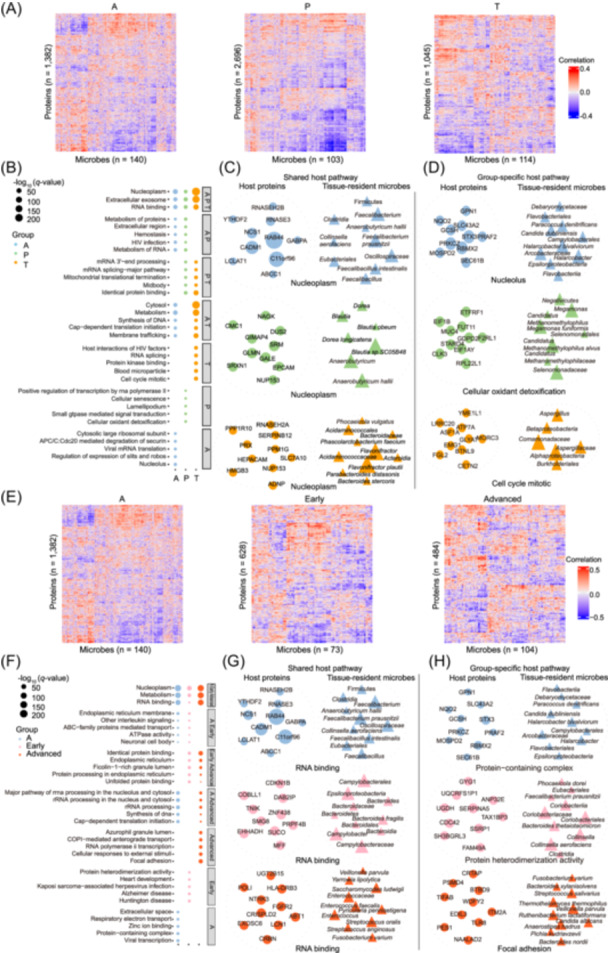
Shared and specific host pathways associated with tissue‐resident microbes through integrated microbiome and proteome analysis. (A) Heatmap shows overall correlation patterns between significantly and robustly selected host genes (rows) and tissue‐resident microbial taxa (columns) identified by the LASSO model in A, P, and T. Correlation coefficients were calculated using Spearman with a robust selection of false discovery rate (FDR) < 0.1. (B) Host pathways enriched with sparse sets of CCA genes associated with microbiome composition in A, P, and T (FDR < 0.1). Only the top three or five significant pathways are shown. Dot size indicates the magnitude of significance of each pathway enrichment, and dot color different groups. (C) Associations between host metabolic pathways shared in A, P, and T and microbial taxa, respectively. The size of the circles and triangles represents the absolute values of the gene and microbial sparsity CCA coefficients, respectively. Microbial taxa belonging to common taxa are shown as overlapping triangles. (D) Associations between host‐specific metabolic pathways (i.e., host pathways for which gene expression correlates with tissue‐resident microbes in only one group) in A, P, and T and microbial taxa, respectively. The size of the circles and triangles represent the absolute values of the gene and microbial sparsity CCA coefficients, respectively. (E) Heatmap shows overall correlation patterns between significantly and robustly selected host genes (rows) and tissue‐resident microbial taxa (columns) identified by the LASSO model in A, early, and advanced. Correlation coefficients were calculated using Spearman with a robust selection of FDR < 0.1. (F) Host pathways enriched with sparse sets of CCA genes associated with microbiome composition in A, early, and advanced (FDR < 0.1). Only the top three or five significant pathways are shown. Dot size indicates the magnitude of significance of each pathway enrichment and dot color different groups. (G) Associations between host metabolic pathways shared in A, early, and advanced and microbial taxa, respectively. The size of the circles and triangles represent the absolute values of the gene and microbial sparsity CCA coefficients, respectively. Microbial taxa belonging to common taxa are shown as overlapping triangles. (H) Associations between host‐specific metabolic pathways (i.e., host pathways for which gene expression correlates with tissue‐resident microbes in only one group) in and A, early, and advanced and microbial taxa, respectively. The size of the circles and triangles represents the absolute values of the gene and microbial sparsity CCA coefficients, respectively.

Previous research indicates that certain microbiota can regulate host gene expression [59]. To systematically investigate such effects, we applied least absolute shrinkage and selection operator (LASSO) penalized regression analysis [17]. This revealed 1382 associations between host proteins and 140 tissue‐resident microbes in A, 2696 in P, and 1045 in T (Figure 6A). Interestingly, while the number of interactions decreased in T compared with P, the strength of associations markedly increased (Figure S6). For instance, *Bacteroides caccae*, a biomarker of inflammatory bowel disease (IBD) [60], showed a positive correlation with *PLCB1* in T, a gene frequently upregulated in cancer [61], but a negative correlation in A and P. Similarly, interactions between *Methanobrevibacter_A smithii_A* and *FBXW9* (a proposed biomarker and therapeutic target in breast cancer) [62] shifted from weak or absent in A/P to significantly positive in T.

Using a machine learning–based multi‐omic integration framework, including sparse canonical correlation analysis and LASSO penalized regression [17], we identified both “shared” and “group‐specific” pathways across the different groups. Among the top shared pathways were the nucleoplasm, extracellular exosomes (implicated in regulation of the TME [63]), and RNA binding pathways (Figure 6B,C). In A, *Faecalibacterium prausnitzii*, a key intestinal species, correlated with host proteins reduced in both IBD and CRC patients, potentially offering anti‐inflammatory benefits [64]. In P, host protein expression was strongly linked with *Anaerobutyricum hallii*, a commensal associated with the reduction of carcinogens in cooked meat [65]. *Flavonifractor plautii*, enriched in young‐onset CRC patients [66], showed significant correlations in T.

We also observed kingdom‐specific interactions. In A, the opportunistic fungal pathogen *Candida dubliniensis* correlated with multiple host proteins (Figure 6D). In P, *Megamonas* correlated with increased risk of colorectal polyps; notably, *Megamonas funiformis* has been proposed as a biomarker for distinguishing post‐cholecystectomy patients from healthy controls [67]. Furthermore, interactions with various archaeal species, such as *Candidatus Methanomethylophilus alvus*, and host proteins were identified. In T, interactions with *Aspergillus*, particularly *Aspergillus sydowii*— previously reported to promote lung cancer development [68]—were observed.

### Shared and distinct signatures of stage‐specific host‐microbiome interactions in CRC progression

Host–microbiome interactions in early tumor tissues (628 associations) exhibited greater complexity than those in advanced tumor tissues (484 associations) (Figure 6E), highlighting the dynamic and stage‐specific nature of host–microbial associations.

Among the shared host–microbe proteins across A, early CRC, and advanced CRC, we observed a notable shift in the interaction between TMED10 and *Bacteroides*, particularly *B. fragilis*, which was positive in A but became negative in advanced CRC (Figure S5D). Additionally, BCL11B showed progressively stronger associations with tissue‐resident microbiota in both early and advanced tumors compared with A.

Our investigation into the top five shared and stage‐enriched pathways across adenoma, early, and advanced tumor tissues revealed three common pathways: nucleoplasm, metabolism, and RNA binding (Figure 6F,G). In early tumors, host proteins were mainly associated with *B. fragilis*, whereas in advanced tumors they were more frequently linked to *Yarrowia lipolytica*. Moreover, in certain pathways, *Candida albicans* was found to interact with diverse host proteins in advanced tumors (Figure 6H), consistent with its reported tumor‐promoting effects [69].

In summary, these findings demonstrate that associations between specific tissue‐resident microbial taxa and host genes or pathways vary substantially across disease states and stages, offering valuable insights into the complex dynamics of host–microbiome interactions during CRC progression.

### Establishing mycotypes through tissue‐resident mycobiome–bacteriome–archaeome–virome–immunome interactions

In the diverse TME, fungal–bacterial–immune clusters exhibit notable variations across different cancer types [19]. These clusters, which may arise through either physical or biochemical mechanisms [70], are hypothesized to co‐occur in A, P, and T, forming distinct mycobiome–bacteriome–archaeome–virome–immunome clusters. Here, the archaeome refers to the archaeal community [71].

**Figure 7:**
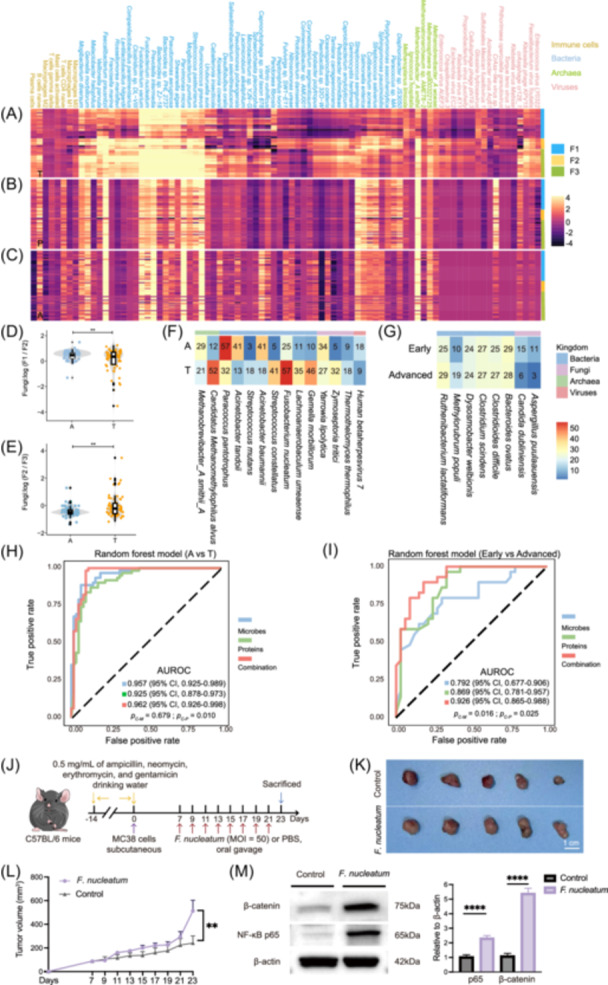
Multi‐kingdom microbiome–host interactions define CRC‐associated mycotypes with functional and clinical implications. (A) Heatmap for inferring conditional probabilities in T. Co‐occurrence analyses of fungal, bacteria, archaeal, viral species, and immune cell compositions using microbe–metabolite vectors (MMvec). Hierarchical clustering information identified three distinct clusters (“mycotypes”) associated with groups of fungal genera: F1, F2, and F3. (B) Heatmap for inferring conditional probabilities in P. Co‐occurrence analyses of fungal, bacteria, archaeal, viral species, and immune cell compositions using MMvec. Hierarchical clustering information identified three distinct clusters (“mycotypes”) associated with groups of fungal genera: F1, F2, and F3. (C) Heatmap for inferring conditional probabilities in A. Co‐occurrence analyses of fungal, bacteria, archaeal, viral species, and immune cell compositions using MMvec. Hierarchical clustering information identified three distinct clusters (“mycotypes”) associated with groups of fungal genera: F1, F2, and F3. (D) Log‐ratios of F1/F2 abundances across A and T. The *p*‐value (two‐sided *t*‐test) was calculated. (E) Log‐ratios of F2/F3 abundances across A and T. The *p*‐value (two‐sided *t*‐test) was calculated. (F) Importance of each listed feature (belonging to the four‐kingdom model) of A and T by the cross‐validation of predictive performance using the internal random forest “Gini importance” method. (G) Importance of each listed feature (belonging to the four‐kingdom model) of early and advanced by the cross‐validation of predictive performance using the internal random forest “Gini importance” method. (H) Receiver operating characteristic (ROC) curves as performance evaluation random‐forest models and to distinguish adenomas from CRC patients. Models were designed based on species (red), or proteins (green), or a combination (blue) of the two features. For the A and T, the species‐based model used 14 species, and the protein‐based model used 8 proteins. Classification accuracy was evaluated on the area under the receiver operating characteristic curve (AUROC) using fivefold cross‐validation testing. *p*_*C‐M*_: *p*_(Combination vs Microbes)_; *p*_*C‐P*_: *p*_(Combination vs Proteins)_. (I) ROC curves as a performance evaluation random‐forest models and to distinguish early and advanced CRC patients. Models were designed based on species (red), or proteins(green), or a combination (blue) of the two features. For the early and advanced tumor tissues, the species‐based model used 8 species, and the protein‐based model used 4 proteins. Classification accuracy was evaluated on the AUROC using fivefold cross‐validation testing. *p*_*C‐M*_: *p*_(Combination vs Microbes)_; *p*_*C‐P*_: *p*_(Combination vs Proteins)_. (J) C57BL/6 mice were gavaged with *F. nucleatum* (MOI = 50, *n* = 5) or PBS (Control) (*n* = 5) once per 3 days in MC38 allograft model. (K and L) Images of tumors, (K) tumor growth curve from *F. nucleatum*‐ or PBS‐treated mice. (M) Western blot analysis of β‐catenin and NF‐κB proteins in MC38 allograft model. Western blot densitometry was shown in the right panel. Data were shown as mean ± SEM. The Two‐way ANOVA (L) and Student's *t* test (M) were used to examine the statistical significance between groups. Scale bar, 1 cm. **p < 0.01. ****p < 0.0001. CI, confidence interval; MOI, multiplicity of infection.

To quantify the conditional probability of each molecule's presence in relation to specific microorganisms, we applied neural network–based microbe–metabolite vectors (MMvec) [72]. This analysis identified three distinct fungal‐driven clusters, termed “mycotypes” (F1–F3). F1 comprised genera such as *Yarrowia*, *Malasseziaceae*, *Saccharomycodes*, and *Candida*. F2 was predominated by *Zymoseptoria*, while F3 encompassed diverse taxa including *Pyricularia* (Table S24). We observed that naïve B cells co‐occurred less frequently with F2 in T and P compared with A, while plasma cells showed increased association with mycotypes during cancer progression. Mycotypes, particularly F3, demonstrated strong co‐occurrence with a range of bacterial taxa such as *Fusobacterium*, *Prevotella*, and *Bacteroides* (Figure 7A–C, Figure S7). Consistent with prior reports [73], the intestinal probiotic *Faecalibacterium prausnitzii* was reduced in tumor tissues (Figure S7).

The co‐occurrence of archaea with mycotypes was broadly similar across groups, whereas significant differences emerged in viral co‐occurrence with F2 and F3 between A and T, suggesting an important role of viruses in cancer progression, consistent with previous results (Figure S2). Finally, log‐ratio comparisons [19] varied between A and T (Figure 7D,E), indicating disturbances in the composition of fungi‐associated microecology. For example, changes in the relative abundance of F1/F2 (comprising *Yarrowia*, *Malasseziaceae*, *Saccharomycodes*, *Candida*, and *Thermothelomyces* vs. *Zymoseptoria*) highlighted ecological reorganization of fungal clusters during CRC progression.

Collectively, these results indicate tissue‐specific mycotypes grounded in multi‐kingdom microbial and host–immune interactions, underscoring ecological reorganization throughout colorectal carcinogenesis.

### Clinical utility of integrated microbial‐protein biomarkers for CRC diagnosis

Alterations in the interaction between gut microbiota and host may provide critical insights into the origins and progression of CRC, offering potential avenues for improved prevention, diagnosis, and treatment. To evaluate their diagnostic potential, we developed a novel strategy integrating tissue‐resident microbial and host protein biomarkers, simultaneously capturing proteomic and microbiome changes.

Using Gini importance ranking from supervised machine learning with random forest (RF) algorithms [24], we identified 8 bacterial, 3 fungal, 2 archaeal, and 1 viral species as candidate biomarkers distinguishing A from T (Figures 7F), and 6 bacterial and 2 fungal species to differentiate early from advanced tumor tissues (Figure 7G). The resulting classifiers achieved an area under the receiver operating characteristic curve (AUROC) of 0.957 (A vs. T) and 0.792 (early vs. advanced) by fivefold cross‐validation (Figure 7H,I). Incorporation of host protein biomarkers into the RF model markedly improved diagnostic performance, with AUROCs of 0.962 (A vs. T) and 0.926 (early vs. advanced), the latter showing a significant increase of 0.134 (*p* = 0.016) (Table S25).

Compared with genomics, proteomic studies of CRC remain limited [74]. According to the central dogma of molecular biology, proteins are the functional products of gene expression and often correlate with RNA levels [75, 76]. Based on this rationale, we collected publicly available transcriptome data to test the performance of our protein biomarkers in CRC screening. Consistent with our findings, the AUROCs of our markers in two external validation cohorts for A/T classifiers were 0.920 and 0.735, respectively, while the early/advanced stage classifier achieved AUROCs ranging from 0.693 to 0.744 (Figure S8).

Distinct correlation patterns were observed between A and T (Figure S9A and Tables S26, S27). For instance, *Acinetobacter baumannii* displayed a negative correlation in A but a positive correlation in T, reflecting its opportunistic pathogenic characteristics. Similarly, both *Fusobacterium nucleatum* and *Streptococcus constellatus* showed significant positive correlations in A but negative correlations in T, suggesting that certain microorganisms present in adenomas may inhibit pathogenic bacterial growth. In early tumors, *Methylorum populi*, *Clostridioides difficile*, and *Ruthenibacterium lactatiformans* exhibited stronger positive correlations, whereas in advanced tumors, *Candida dubliniensis* shifted from negative in early CRC to positive correlations, indicating progressive enrichment of pathogenic microorganisms (Figure S9B and Tables S28, S29).

### Validation of *Fusobacterium nucleatum*‐induced Wnt and NF‐κB pathway activation in vivo

Among the microbial biomarkers we identified, *F. nucleatum* exhibited high abundance in T and a stage‐dependent correlation pattern (Figure 2E, Figure S9), suggesting an active role in CRC progression. To functionally validate its association with the Wnt and NF‐κB pathways observed in our multi‐omics analysis, we established a CRC allograft mouse model by subcutaneous implantation of murine MC38 cells into C57BL/6 mice. Compared with PBS controls, *F. nucleatum*‐treated mice exhibited increased tumor size (Figure 7J–L). Western blot analysis confirmed upregulation of β‐catenin and NF‐κB p65 in tumors from *F. nucleatum*‐infected mice (Figure 7M), directly linking *F. nucleatum* to activation of these oncogenic signaling pathways. These in vivo results align with our proteomic data (Figure 4B) and reinforce the role of *F. nucleatum* as a microbial modulator of CRC progression [77, 78, 79, 80, 81, 82].

Taken together, our results demonstrate that paired tissue‐resident microbial taxa and host proteins can serve not only as diagnostic biomarkers to differentiate CRC states and stages, but also as active modulators of oncogenic pathways. These findings provide a comprehensive framework for understanding state‐ and stage‐specific host–microbiome interactions in colorectal carcinogenesis and highlight their translational potential in clinical applications.

## DISCUSSION

Colorectal cancer urgently requires effective biomarkers to enable efficient screening and treatment strategies, given the heterogeneity of patient outcomes and the variable efficacy of existing therapies [83, 84]. The intratumoral microbiome is increasingly recognized for its role in modulating tumor progression and influencing immunotherapy response through diverse mechanisms, including the production of genotoxins, metabolic modulation (e.g., butyrate, secondary bile acids), and regulation of local immune surveillance [15, 31, 85, 86, 87, 88, 89, 90, 91]. Consequently, specific microbial signatures are emerging as promising independent prognostic and predictive biomarkers [18, 86, 87, 90, 92].

Although numerous studies have associated gut microbial composition or host transcriptomic signatures with CRC development [93, 94], direct investigations of intratumoral host–microbiome interactions at the protein level remain limited. This knowledge gap is particularly critical, as posttranslational regulation may decouple protein abundance from gene expression, and many microbial effects are mediated through direct protein–pathway interactions [95, 96, 97, 98, 99].

To bridge this gap, we applied a machine learning‐based multi‐omic framework integrating deep metagenomic sequencing and proteomics—an approach well suited to uncovering novel, biologically relevant interactions from high‐dimensional data [17]. This strategy enabled us to map both shared and distinct interplays between tissue‐resident microbiota and host proteins across A, P, and T tissues.

Together, these results underscore the power of integrated multi‐omics profiling to uncover robust biomarker candidates, with implications for both mechanistic understanding and clinical translation in precision oncology.

Our multi‐kingdom analysis reveals distinct ecological shifts across fungal, bacterial, archaeal, and viral communities during CRC progression, consistent with recent research [18, 19]. Specifically, *Bacteroides* and *Fusobacterium* were notably enriched in T compared with A, reflecting patterns observed in gut microbiome studies [20, 36, 57]. In P, *Bacteroides caccae* and *Bacteroides cellulosilyticus* showed strong negative correlations with most pathways, indicating their potential as diagnostic biomarkers of CRC [100, 101]. Altered gut microbiota, including *F. nucleatum* and *Parvimonas micra* [20, 81, 102, 103], were consistently implicated in CRC development.

Fungal alterations included a significant increase in *Zymoseptoria tritici* and a decrease in *Yarrowia lipolytica* in P and T compared with A (Figure 2E,F). While *Z. tritici* is an agricultural pathogen [104] also detected in human stool and tissues [18, 20, 30], *Y. lipolytica* is enriched in breast tumors [18] and recognized for its potential to produce health‐beneficial metabolites such as resveratrol [105]. Similarly, *Candida albicans* was less prevalent in P and T compared with A, while other fungi were largely conserved between P and T, supporting the idea that adjacent tissues share more similar microenvironments with tumors (Figure 2B).

Archaeal analysis identified *Methanobrevibacter smithii* as highly abundant across A, P, and T (Figure 2C), consistent with reports that *M. smithii* dominates the archaeal virome when infected by viruses [106]. Notably, enhanced positive correlations were observed in P and T compared with A, possibly reflecting interactions between CRC‐enriched fungi [30] and the accumulation of pathogenic microorganisms, which alter the TME [57, 81]. Viral profiling revealed 374 viruses, with distinct co‐occurrence patterns involving fungal clusters (F2, F3) across A and T (Figure 7A) and stage‐specific shifts (Figure 2D), suggesting potential viral involvement in CRC progression through multiple mechanisms. The virome, an essential functional component of the microbiome, influences microbial ecology and cancer progression through both bacteriophage–bacteria interactions [107, 108, 109] and direct oncogenic mechanisms, including chronic inflammation and immune evasion [110, 111].

Our multi‐kingdom microbial interaction analysis further revealed distinct ecological dynamics during colorectal carcinogenesis. The weaker microbial correlations observed in A, contrasted with stronger negative correlations in T (Figure 3B, Figure S3A), likely reflect competitive exclusion driven by pathogen enrichment. This niche competition may result from specific carcinogenic pathogens [37, 112]; for instance, *F. nucleatum* may suppress commensals via virulence factors (e.g., FadA, Fap2) to secure its niche [44, 113, 114], while ETBF employs bacteriocins to eliminate competitors [115]. These processes concurrently activate host oncogenic pathways such as Wnt/β‐catenin and NF‐κB [51, 80, 82, 116, 117, 118, 119, 120, 121]. Focused analysis of Ascomycota revealed stage‐specific interaction shifts: stronger negative correlations with Firmicutes from adenoma to advanced CRC and emerging positive correlations with Proteobacteria in CRC (Figure S3B). These observations suggest that CRC‐associated pathogens reshape niche availability, aligning with emerging evidence that fungal–bacterial interactions play crucial roles in CRC pathogenesis [52, 122, 123, 124].

Proteomic profiling revealed widespread dysregulation across multiple cancer hallmark‐related signaling pathways, consistent with previous findings [49]. A key finding was the significant activation of the Wnt/β‐catenin and NF‐κB signaling pathways (Figure 4B), both well‐established drivers of CRC progression [50, 51, 78, 125, 126, 127]. Activation of these pathways may be mechanistically linked to the observed enrichment of microbiota such as *F. nucleatum* and ETBF in tumor tissues. Indeed, ETBF infection in organoids increased Ki67 and phosphorylated β‐catenin (Figure 4F,G), while *F. nucleatum* in an allograft mouse model upregulated β‐catenin and NF‐κB signaling (Figure 7J–M), validating their roles as microbial modulators of oncogenic pathways [38, 52, 79, 80, 81, 82].

Proteomic subtyping identified four CRC subtypes (C1–C4) with the potential to guide personalized treatment (Figure 4C). Among these, the C4 subtype was characterized by reduced metabolic activity and Hippo pathway signaling, and it exhibited the highest TIDE score (Figure 4E), suggesting an immunosuppressive microenvironment and poorer response to immune checkpoint therapy [128, 129, 130]. Mechanistically, metabolic competition and Hippo pathway effectors—which upregulate *PD‐L1* expression and activate the IL‐6/STAT3 pathway—may drive this phenotype [131, 132, 133]. The positive correlation between *P. intermedia* and TIDE in C4 further supports its role in immune modulation. *P. intermedia* may contribute to immune suppression via pathways such as TLR4/NF‐κB activation, as its lipopolysaccharide (LPS) is a known agonist of this pathway and a potent inducer of pro‐inflammatory cytokines and nitric oxide—key mediators of an immunosuppressive microenvironment [134, 135, 136, 137, 138, 139, 140, 141]. Furthermore, NF‐κB is an established transcriptional regulator of *PD‐L*1 expression [142, 143, 144], and related periodontal pathogens such as *Porphyromonas gingivalis* have been shown to upregulate *PD‐L1* in tumor cells [145, 146]. Its co‐occurrence with *F. nucleatum* in association with *TP53* mutations also suggests potential synergistic effects in immune modulation [147].

Differences in protein–protein interactions and pathways in tumor tissues provide further insights for CRC research. For example, the senescence pathway has been implicated in breast cancer development by acting as a barrier to excessive cell growth and as a regulator of tumor initiation and progression [148]. IL‐6 is known to activate autophagy and contribute to chemoresistance in CRC [149], while the 14‐3‐3 pathway is integral to cancer stress adaptation [150] (Figure 5B). Our results revealed differential expression factors and regulatory networks across tissues, suggesting host proteins as potential targets for CRC diagnosis and treatment.

Using a machine learning‐based multi‐omic framework [17], we identified shared and specific host–microbiome interplays. Intriguingly, different microbes corresponded with various host proteins within the same pathway, thereby influencing disease‐specific pathophysiological processes [17]. Specific host protein–microbe associations were detected across A, P, and T. For example, in tumors, the cell cycle mitotic pathway—associated with *Aspergillus*, known to promote CRC [30]—illustrates how intratumoral microbiota regulate host protein expression in specific disease states. Furthermore, our analysis showed that the correlation between TMED10 and the microbiome gradually weakened from A to early and advanced tumor stages, while the correlation between BCL11B and the microbiome progressively increased (Figure S5D). This may be explained by TMED10's role as a biomarker of favorable prognosis in prostate cancer [151] and BCL11B's function as a tumor suppressor [152, 153]. These findings warrant further investigation into their specific interactions with microbiota.

Finally, our RF models integrating microbiome and protein features achieved high AUROC scores in distinguishing early from advanced tumor tissues, underscoring the additive predictive value of multi‐omic features.

While proteomics studies in CRC are less extensive than transcriptomics, we used public transcriptome data to validate our protein‐based findings. Our study focused on proteins and combined microbiome and proteome data from tumors to investigate host–microbiota interactions. We showed that integrating microbial and protein markers improves the detection of CRA and cancers and helps distinguish early from advanced tumors. Although microbiota alone was not sufficient for diagnosis, adding protein data significantly improved accuracy. Understanding how microbiota and host proteins interact in tumors could ultimately lead to precision treatments for microbiota‐related diseases.

We acknowledge that our findings represent observational associations that require further mechanistic validation. Future studies should incorporate metabolomics with spatial transcriptomics or proteomics to map host–microbiome interactions and microbial metabolite–host pathway crosstalk (e.g., short‐chain fatty acids, deoxycholic acid) in the TME, providing mechanistic depth to metabolic alterations in CRC and aiding novel therapeutic target discovery [154, 155, 156, 157, 158, 159, 160, 161, 162, 163, 164, 165, 166]. Such efforts must also account for confounders such as tumor location and dietary habits, which significantly modulate microbial composition and metabolite production [162, 167, 168, 169, 170, 171, 172, 173, 174]. In addition, microbial contributions to treatment resistance and the role of phages and tumor‐associated viruses warrant systematic exploration [107, 108].

Finally, relying on transcriptomic data for external validation of protein biomarkers is a limitation, as the correlation between transcripts and proteins can vary depending on biological context and posttranslational regulation. Establishing more cohorts for proteomic validation will therefore be critical. Although fecal microbiota analysis has gained global recognition as a minimally invasive approach, interindividual variability limits its predictive power [30, 122, 175]. Our results demonstrate that combining tissue‐resident microbial signatures with host protein biomarkers significantly enhances diagnostic accuracy, thereby validating the synergistic potential of multi‐omics approaches in precision anticancer diagnosis.

Thus, developing hybrid models that combine intratumoral and fecal microbial biomarkers may ultimately merge the precision of tissue analysis with the accessibility of fecal testing to optimize CRC screening. These integrated approaches, with clear translational potential, could be particularly valuable for longitudinal monitoring and population‐based screening programs.

## CONCLUSION

This study highlights the clinical and biological importance of intratumoral microbiome–host interactions in colorectal carcinogenesis. By integrating deep intratumoral metagenomics with host proteomics, we systematically characterized multi‐kingdom microbial ecosystems and proteomic alterations across the adenoma–carcinoma sequence. We identified combined microbial–protein signatures capable of distinguishing adenoma from carcinoma and early‐ from advanced‐stage CRC, achieving strong diagnostic performance. Functional validation in organoids and allograft models confirmed that ETBF and *F. nucleatum* promote tumor growth by activating Wnt/β‐catenin and NF‐κB signaling.

Collectively, our findings underscore pathogenic microbes as potent modulators of CRC progression and provide a foundation for microbiota‐based diagnostics and targeted therapeutics. These insights may help reduce the clinical and socioeconomic burden of CRC by advancing precision strategies for early detection and intervention.

## METHODS

### Study populations

#### CRC patients

A total of 69 individual patients scheduled for colonic resection in the surgery room at Renji Hospital, Affiliated to Shanghai Jiao Tong University School of Medicine, were recruited at an initial CRC diagnosis for this study. Inclusion criteria were: no prior history of CRC, IBD, or irritable bowel syndrome to exclude confounding inflammatory/malignant conditions; no antibiotic use within 1 month before surgery; no proton pump inhibitor use for at least 2 weeks before sampling; no neoadjuvant therapy; and pathologically confirmed colorectal cancer. Exclusion criteria included missing detailed clinical or pathological information. After applying these criteria, 62 patients with confirmed pathological and clinical diagnosis of CRC were enrolled. Pathological diagnosis was performed by experienced pathologists using hematoxylin and eosin‐stained slides in accordance with the 2005 World Health Organization histological classification. Demographic and clinical information, including age, sex, BMI, and tumor location, were recorded for each patient. All patients provided written informed consent for participation in this study.

#### CRA patients

A total of 62 patients with endoscopically confirmed adenomatous polyps were recruited Renji Hospital Affiliated to Shanghai Jiao Tong University School of Medicine were recruited for the study. Written informed consent was obtained from all participants before data and specimen collection. Recruitment criteria included patients with ultimately endoscopically confirmed adenomatous polyps among individuals presenting with gastrointestinal symptoms in the gastroenterology clinic or undergoing screening colonoscopy. Exclusion criteria were identical to those applied to CRC patients.

### Sample collection

#### CRC patients

All patients underwent surgery in a fasting state. The paired tumor/para‐tumor tissues were collected from pathologically confirmed diagnosis of colorectal cancer patients. The para‐tumor tissues were collected >5 cm from the tumor under sterile conditions, near the surgical resection margins. Each sample from individuals was divided into three equal parts. One was placed in 1 mL RNAlater (QIAGEN, Germany ID: 76106) and stored at −80°C, one was placed in 4% paraformaldehyde (Beyotime, China P0099‐500 mL), stored at 4°C for 24 h, and then embedded with paraffin (FFPE), and one was stored directly at −80°C. To minimize contamination risks, during the sample collection phase, immediately following surgical excision by clinicians, all samples were aseptically collected and transported via a cold chain system under strictly anaerobic conditions.

#### CRA patients

Up to two mucosal biopsies were obtained per individual during endoscopy after bowel preparation. The samples were stored at −80°C immediately or made into FFPE as described above. Sample processing details were as above.

#### Environmental controls

Although both the surgery and endoscopy room are relatively clean, they are not completely sterile, such as dust, air, and human commensals. To avoid the various sources of contamination from the hospital, it is essential to set up proper environmental controls. From the beginning of surgery or endoscopy, several 2 mL test tubes with sterile PBS were placed in the sampling environment until the end of surgery or endoscopy, and stored along with the tissue samples at −80°C.

#### DNA extraction controls

As contaminations in the reagents are difficult to eliminate, DNA Extraction controls (EXC) were the tube with sterile PBS introduced during DNA extraction from the laboratory environment.

### Staining methods

#### Fluorescence in situ hybridization (FISH)

The D223 28S rRNA gene probe labeled with the 5'Cy3 fluorophore (extinction wavelength, 555 nm; emission wavelength, 570 nm; Molecular Probes, Eugene, OR) was used to detect the fungal colonization within human tissues by FISH. Fluorescence microscopic analysis was conducted with Nikon Eclipse 90i confocal microscope (Nikon, Melville, NY) using a Cy3 labeled‐probe at 350 pmol/mL as previously described [176].

#### Hematoxylin‐eosin (HE) staining

The slides were immersed into xylene I 20min‐xylene II 20min‐absolute ethanol I 5min‐absolute ethanol II 5min‐75% alcohol 5 min in turn, and washed with tap water. Tissue sections were stained with hematoxylin staining solution for 3–5 min, washed with tap water, differentiated with differentiation solution, washed with tap water, returned to blue with blue solution, and rinsed with running water. They were then dehydrated in 85% and 95% graded alcohol for 5 min, and stained in eosin for 5 min. The tissue slices were placed in anhydrous ethanol I for 5 min ‐ anhydrous ethanol II for 5 min ‐ anhydrous ethanol III for 5 min ‐ dimethyl I for 5 min ‐ xylene II for 5 min to be transparent, and sealed with neutral gum.

#### Polymerase chain reaction (PCR)

PCR was performed using Eppendorf Mastercycler Nexus gradient PCR. Primers for 16S rRNA are listed as follows: F: GTGCCAGCMGCCGCGGTAA; R: GGACTACHHVGGGTWTCTAAT. Primers for ITS2 are listed as follows: F: ACACACCGCCCGTCGCTACT; R: TTTCGCTGCGTTCTTCATCG.

### DNA extraction and sequencing analysis

#### DNA extraction

Tissue samples were transported on dry ice and immediately transferred to a −80°C freezer until use. They were rinsed with sterile PBS at least three times to avoid fecal contamination. During DNA extraction, all extraction personnel wore full personal protective equipment (gloves, masks, gowns) to prevent skin contamination, and work surfaces were UV‐treated before extractions. The entire procedures were conducted in strict accordance with laboratory protocols under sterile conditions within a Class II biosafety cabinet, with surfaces routinely disinfected using 75% alcohol and other fungicidal agents. Additionally, we collected parallel environmental samples from the operating room during sample collection and negative controls during DNA extraction. Genomic DNA was extracted using the Qiagen DNeasy Blood and Tissue Kit (Cat no. 69506, Valencia, CA) with the following modifications: the tissue samples were pretreated by lysis buffer overnight and then beads‐beating with glass beads (Cat no. G8772, acid‐washed, Sigma) on homogenizer (FastPrep‐24 5G, MP Biomedicals) to get a better fungi‐enriched DNA yield [177]. The total amount, purity, and integrity of DNA were analyzed using Agilent 5400. Both sample quality and concentration meet the quality requirements of library construction and sequencing.

#### Library construction and high‐throughput sequencing

A total amount of 1 μL DNA per sample was used for the DNA sample preparations. Sequencing libraries were generated with NEBNext Ultra™ DNA Library Prep Kit for Illumina (NEB, USA) following the manufacturer's instructions. In detail, the DNA sample was fragmented to a length of about 350 bp, DNA fragments were end‐polished, A‐tailed, and ligated with the full‐length adaptor for Illumina sequencing with further PCR amplification. The library quality was confirmed with an Agilent 2100 Bioanalyzer and quantified using real‐time PCR. The clustering of the index‐coded samples was performed on a cBot Cluster Generation System. The library preparations were sequenced by Illumina NovaSeq 6000 and paired‐end reads were generated to a targeted data set size of 20 GB.

### Extraction of proteins and nano‐UHPLC‐MS/MS analysis

#### Protein extraction and digestion

Tissue samples were lysed in SDS lysis buffer for 20 min, followed by boiling at 100°C for 5 min. Protein samples are added to iodoacetamide (IAA) at a final concentration of 200 mM for alkylation of 1 h in the dark. Then, five times the volume of precooled acetone was added, and the protein was precipitated in a refrigerator at −20°C overnight. Finally, the protein precipitates were decomposed and digested by trypsin (Promega) in a 50:1 protein‐to‐enzyme ratio. Trypsin peptides, collected by centrifugation at 14,000 *g* for 20 min, were treated with 1% trifluoroacetic acid (TFA), purified with C18Ziptips, eluted in 0.1% TFA in 50 to 70% acetonitrile, dried by speed‐vac (ThermoSavant), and resuspended in 1% formic acid 5% acetonitrile. The iRT standard peptides (Biognosys, Schlieren, Switzerland) are added to the sample before analysis according to the manufacturer's instructions.

#### Nano‐UHPLC‐MS/MS analysis

The peptide was resolved in 0.1% formic acid (buffer A) and analyzed with the FAMES interface of the Orbitrap Exploris 480 mass spectrometer in combination with the EASY‐nanolc1200 system (Thermo Fisher Scientific, USA). The 2 μL peptide sample was loaded onto a 20 cm analytical column (75 μm inner diameter, 1.7 μm resin (waters BEH)) and separated with 120 min‐gradient starting at 6% buffer B (80% ACN with 0.1% FA) followed by a linear gradient to 20% in 99 min, 32% in 5 min, 80% in 1 min and stayed there for 5 min. The column flow rate was maintained at 250 nL/min with the column temperature of 55°C. The electrospray voltage was set to 2 kV. The mass spectrometer was run under data‐independent acquisition mode with a hybrid data strategy. A survey scan was acquired at 120,000 resolution, normalized AGC target of 3e6 and a maximum injection time of 20 ms. In the DIA MS2 acquisition, variable Isolation window was performed with window widths of 30 m/z (mass range from m/z 350‐408 with 2 windows), 10 m/z (mass range from m/z 408‐795 with 43 windows), 20 m/z (mass range from m/z 795–985 with 11 windows) and 50 m/z (mass range from m/z 985–1200 with 4 windows). One full scan followed by 20 windows with a resolution of 30,000, normalized AGC target of 1e6, and normalized collision energy stepped at 27, 30, and 33. Compensation voltage (CV) of −45 and −65 V were selected and applied to MS/MS scans and the corresponding survey scan. Raw Data of DIA were processed and analyzed by Spectronaut 14 (Biognosys AG, Switzerland) with default settings. The MS raw data were searched against the human UniProt fasta database within the default parameters.

### Preprocessing and analysis of metagenomic data

#### Sequence quality control and contaminant removal

We addressed potential contamination issues using a three‐step approach: 1. Including sequencing controls. These included measures in the hospital and laboratory settings, stringent controls on sample handling and processing, and meticulous DNA extraction procedures. 2. Reviewing the literature to exclude potential contaminants (as shown in Table S2) [33, 178, 179, 180, 181, 182, 183, 184, 185]. 3. Modeling the prevalence of taxa in control samples to identify contaminants using the *Decontam* package [29] by using the combined method under the default parameter (probability threshold = 0.1). The KneadData (https://github.com/biobakery/kneaddata) v.0.10.0 tool was used to ensure that the data consisting of high‐quality microbial reads were not affected by contaminants. The Trimmomatic (v0.39) (SLIDINGWINDOW:4:15 MINLEN:75) was used to remove low‐quality reads. Next, the remaining reads were mapped to the animal genome (GRCh38, F.catus_Fca126, canFam6, mm39, rn7, susScr11, GalGal6 and bosTau9; UCSC Genome Browser) and 42,099 bacterial plasmids (National Center for Biotechnology Information (NCBI) RefSeq database accessed in January 2022), 8265 complete plastomes (NCBI RefSeq database accessed in January 2022) and 6093 UniVec sequences (NCBI RefSeq database accessed in January 2022) by bowtie2 v.2.3.5.1. The matched reads were removed as potentially host‐ and laboratory‐associated sequences.

#### Microbial taxonomic profiling

Taxonomic classification of bacteria, fungi, archaea, and viruses was assigned to metagenomic reads with Kraken2(2.1.2), an improved metagenomic taxonomy classifer that utilizes k‐mer‐based algorithms. A custom database consisting of 72,441 bacterial, 773 archaeal, and 14,791 viral reference genome sequences from the NCBI RefSeq database (accessed in January 2022) and 1678 fungal reference genome sequences from the NCBI RefSeq database (accessed in January 2022) and 1167 archaeal reference genomes from Chibani CM [186] was built using Kraken2. Bracken (v2.5.0) was used to accurately estimate taxonomic abundance, particularly at the species and genus levels based on Kraken2. To remove the potential contaminants, we performed the *Decontam* R package [29] by using the combined method under default parameter (probability threshold = 0.1), which had the cleanest bimodal distribution by combining the frequency‐based and prevalence‐based scores into a composite score. The read counts and relative abundance of species after decontamination were used for further analysis (Tables S2–S8).

#### Functional profiling

The above high‐quality reads were preprocessed and assembled into contigs with Megahit v1.2.9 using “meta‐sensitive” parameters; contigs less than 500 bp were removed from further analysis. Prodigal v.2.6.3 was used to predict genes via the metagenome mode (‐p meta). A nonredundant microbial gene reference sequence was constructed using CD‐HIT (v4.8.1). The threshold of sequence identity was 0.95, and the minimum coverage threshold of shorter sequences was 0.9.

The reference was annotated with EggNOG mapper v2.1.8 based on EggNOG orthology data. Moreover, gene abundance was estimated by mapping high‐quality reads to reference sequences using CoverM v0.6.1 (https://github.com/wwood/CoverM). An index was created against contigs from the nonredundant genes generated by the Burrows–Wheeler Aligner (BWA, v0.7.17‐r1188). Clean reads were then mapped to the contig index (BWA MEM), and SAM files were converted into BAM files with SAMtools (v1.15.1). Then, the CoverM was used to calculate the coverage of genes in the original contigs (coverm contig). The relative abundances of EggNOG genes, KEGG KO groups or description were estimated by summing the relative abundances of genes annotated to belong to the same KOs or descriptions.

#### Differential species identification

Linear discriminant analysis effect size (LEfSe) was used to identify differentially abundant microbes between groups at the species lever. The screening criteria were *p* < 0.05 and linear discriminant analysis (LDA) value. The top 30 bacterial species with LDA ≥ 2 were selected, while all species in Fungi/Archaea/Viruses were selected with LDA ≥ 1.

#### Microbial communities division analysis

The species with the deepest taxonomic annotation was fitted to the DMM models, and the microbial communities were divided into a finite number of clusters with the Laplace approximation. Fitting microbiome data to DMM models defined four groups of A, P, and T at order level, and the species differences were analyzed by LEfSe at the species, genus, and family level. In the four groups, the top 5 bacteria with *p* < 0.05 and LDA ≥ 2, and the top 2 fungi, archaea, and viruses with LDA values were screened, respectively. We did the lefse analysis on the data, adjusting the parameters to ensure that the output contained all *p*‐values and “FDR” corrected *p*‐value results. We found that the number of entries with *p*.adj < 0.25 in the T versus P was 1, and the number of entries with *p*.adj < 0.25 in the early/advanced stages was 0. Filtering for differentiated species according to the corrected *p*‐value would have resulted in the loss of a large amount of useful information for us, so we decided to filter the results using *p*.unadj < 0.05. The species selected for each group has a greater average abundance in that group than in the other groups. A total of 24 species were obtained and visualized by a heatmap.

#### Correlation analysis with SparCC algorithm

The SparCC algorithm [42] was used to analyze the interaction of microorganisms in each group at the species and genus levels. The parameters were set as xi = 50 and ni = 100.

#### Co‐occurrence analyses with MMvec

The *Bioinformatics* package in R was used to implement MMvec analysis. To explore the co‐occurrence clustering, hierarchical clustering was performed by using the results of T group and identified the three fungi‐driven “mycotypes” (F1/F2/F3). ANOVAR analysis was then performed on immune cells, bacteria, archaea, and viruses, respectively, based on mycotypes, and entries with *p* < 0.05 were selected for comparing in pairs. The differential entries between two mycotypes were determined in accordance with (i) the *p*‐value and Median difference between each mycotypes and other mycotypes, (ii) *p* < 0.05, (iii) Median > 0, and (iv) Median (bacteria > 1.6, archaea > 0.7, viruses > 0.9, immune cells > 0.3). The ratio of mycotypes was calculated by log (x+1) transformation.

### Preprocessing and differential protein expression analysis

#### Subtyping analysis using proteomic data

For proteomic subtype prediction, we applied consensus clustering on 2847 DEPs between 62 primary tumors and paired nontumor tissues using hierarchical clustering. In addition, we made an attempt to calculate the expression distance by using the *APE* package in R, and then constructed the phylogenetic tree by using the neighbor‐joining (NJ) method. The result shows a high degree of similarity between the tree and hierarchical clustering.

And the quantitative analysis data achieved by the current technology is more focused on the acquisition of molecular typing rather than the acquisition of evolutionary relationships for comparison with published literature. Thus, we finally adopted the existing hierarchical clustering method. And it was also aligned with the consensus molecular subtypes (CMS 1‐4) by the CMSclassifier (https://github.com/Sage-Bionetworks/CMSclassifier) and the CPTAC subtypes (ProS A‐E) using predefined signature. Functional annotation of DEPs was performed using gene set enrichment analysis using KEGG and the hallmarks of cancer gene sets database of the molecular signatures database (MSigDB). The regulatory network of differential expressed proteins was analyzed using ingenuity pathway analysis (IPA, QIAGEN Inc., https://www.qiagenbioinformatics.com/products/ingenuity-pathway-analysis). Canonical signaling pathways enriched by the DEP were identified and rated according to *p*‐values. The *z*‐scores of significantly involved canonical signaling pathways were also determined. *Z*‐scores were presented by orange or blue colors [187]. Functional co‐expression network analysis was used to identify the protein modules that were statistically co‐expressed [188].

#### Integrated analysis of intratumor microbiome and host proteome

We used the Sparse canonical correlation analysis (Sparce CCA) and the LASSO machine learning framework for integrating intratumor microbiome and host proteome data [17]. Significant host–microbiome interactions were identified using a false discovery rate (FDR) threshold of 0.1, consistent with prior exploratory studies in high‐dimensional microbiome–host integration [17]. For each host gene, we performed leave‐one‐out cross‐validation for parameter tuning and iteratively estimated the error term (σ) to ensure robustness. This analytical framework was applied independently to five clinical subgroups (A, T, P, early‐stage, and advanced‐stage) to evaluate the consistency of interactions across different biological contexts.

### Bacterial strain and culture

*F. nucleatum* was purchased from American type culture collection (ATCC, *Fusobacterium nucleatumsub*sp.nucleatum ATCC 25586). Enterotoxigenic *Bacteroides fragilis* (ETBF) was a generous gift (ATCC 43860) from Professor Jie Hong at Renji Hospital Affiliated to Shanghai Jiao Tong University School of Medicine. *F. nucleatum* and ETBF were cultured at 37°C under anaerobic conditions in Gifu Anaerobic Medium, Modified (HB8518‐3) supplemented with 1% vitamin K1 and hemin chloride.

### Organoid establishment and culture

#### Human tissues

Tissue material was obtained from Shanghai Tenth People's Hospital with informed consent, and the study was approved by the ethical committee of Shanghai Tenth People's Hospital. All patients were pathologically diagnosed with colorectal cancer.

#### Patient‐derived organoid culture

Patient‐derived tumor colorectal organoids were established and maintained as described from isolated colonic epithelium [189, 190, 191]. In brief, long‐term tumor colonic organoids were cultured in human intestinal stem cell medium (HISM) composed of advanced DMEM/F12 (Gibco) with penicillin/streptomycin, 10 mM HEPES, 1 GlutaMAX, 1B27 (Invitrogen) and 1 μM N‐acetylcysteine (SIGMA), supplemented with 50 ng mL−1 human recombinant EGF (Peprotech), 100 ng mL−1 human recombinant Noggin (Novoprotein), 0.5 μM A83‐01 (Tocris), 10 nM Gastrin (Sigma), 10 mM nicotinamide (Sigma), 10 nM prostaglandin E2 (MCE), and R‐Spondin1‐CM (10% final concentration). Tumor organoids were passaged every 5–7 days. Organoids were mechanically dissociated by 10–15× repeated pipetting using a 1 mL pipette tip and seeded in Basement Membrane Extract (BME) (R&D Systems).

#### PDO and bacteria coculture experiment and immunostaining analysis

PDOs were seeded into 8 Chambered Coverglass system (Cellvis) at a density of 300 organoids per well when passaged. After 48 h of culture, the medium was replaced with fresh HISM containing ETBF (MOI = 50) for 4 h coculture at 37°C, and subsequently fixed 15 min in 4% PFA for immunostaining analysis. Next, organoids were permeabilized and blocked in PBS containing 0.5% Triton X‐100 and 5% normal goat serum for 1 h at room temperature. Organoids were incubated overnight at 4°C in blocking buffer containing primary antibodies. Primary antibodies used were polyclonal rabbit anti‐Ki67 (1:300, Thermo Fisher, PA5‐114437), rabbit anti‐active β‐catenin (1:300, Cell Signaling Technology, 8814). Organoids were incubated with Alexa 555 conjugated anti‐rabbit secondary antibodies (1:500, Invitrogen) in blocking buffer containing DAPI (1:1000, Invitrogen) for 2 h at room temperature. Images were captured with an SP8 confocal microscope (Leica).

### Animals and in vivo procedures

All mice were bred and maintained under specific pathogen‐free conditions. Groups of male C57BL/6 mice, aged 6–8 weeks, were purchased from Zhejiang Vital River Laboratory Animal Technology Co., Ltd.

To ablate the microbiome in mice, animals were administered by drinking water containing 0.5 mg/mL of ampicillin (MeilunBio, MA0317), neomycin (TargetMol, T0950), erythromycin (TargetMol, T1032), and gentamicin (TargetMol, T25447) for 14 days [192].

To established a CRC allograft model, age‐matched male mice were subcutaneously injected with 5 × 10^5^ MC38 cells embedded in Matrigel (Corning, 354248). Mice were randomly assigned to different experimental groups, where the treatment group was administered *F. nucleatum* with a multiplicity of infection (MOI) of 50 [2.5 × 10^7^ colony‐forming unit (CFU)] by gavage, whereas the control group was given equal amounts of phosphate‐buffered saline (PBS) synchronously every 2 days. Tumor volume was recorded every 3 days using a vernier caliper (tumor volume = 1/2 × length × width^2^). Tumor‐bearing mice were monitored regularly for reduced feeding, weight loss, or dehydration.

### Western blot

Proteins were extracted from tumor tissues using radio immunoprecipitation assay (RIPA) lysis buffer (Beyotime, P0013B) supplemented with phenylmethanesulfonyl fluoride (PMSF, Beyotime, ST506), a protease inhibitor cocktail (Sigma, 11836170001), and phosphatase inhibitor cocktail A (Beyotime, P1081). Protein concentration was determined by Pierce™ BCA Protein Assay Kit (Thermo Scientific, 23227). Samples were resolved using a 10% sodium dodecyl sulfate‐polyacrylamide gel electrophoresis system and transferred onto polyvinylidene difluoride (PVDF) membranes. The PVDF membranes were blocked in 5% skimmed milk (BD, 232100) in TBST for 1 h and incubated with primary antibodies overnight at 4°C. The primary antibodies used included polyclonal rabbit anti‐β‐catenin (1:1,000, Proteintech, 51067‐2‐AP), polyclonal rabbit anti‐NF‐κB p65 (1:1000, Proteintech, 10745‐1‐AP) and mouse anti‐beta Actin mAb(1:5000, abcam, ab6276). After washing with TBST, membranes were incubated with the appropriate HRP‐linked secondary antibodies (anti‐rabbit IgG, 1:5000, Cell Signaling Technology, 7074; or anti‐mouse IgG, 1:5000, Cell Signaling Technology, 7076) for 1 h at room temperature. Following further washes, proteins were visualized using SuperSignal™ West Femto Maximum Sensitivity Substrate (Thermo Scientific, 34096) and SuperSignal™ West Pico PLUS Chemiluminescent Substrate (Thermo Scientific, 34577) in a chemiluminescence imaging system BG‐gdsAUTO 720 (Baygene, China). The experiments were independently repeated at least three times to ensure reproducibility. Data were analyzed using ImageJ software, where the grayscale value of each target protein band was normalized to that of its corresponding internal reference.

### Statistical analysis

#### Microbial ecological analysis

The analysis of alpha diversity and beta diversity was performed using R v4.3.0 *vegan* package and *ecodist* and *vegan* package, respectively. Differences in Shannon and Simpson's index across groups were assessed using the Kruskal–Wallis test, followed by post hoc Wilcoxon rank‐sum tests with Benjamini–Hochberg correction when significant differences were detected (*p* < 0.05). In addition, *p* values of beta diversity based on Bray–Curtis distance were calculated by permutational multivariate analysis of variance (PERMANOVA) (two‐sided test) with 999 permutations. Chi‐square test was used for sex and *t*‐test was used for age and BMI. *p* < 0.05 was statistically significant.

#### Differential host protein analysis

DEPs of tumor (T) and para‐tumor (P) were assessed by a paired *t*‐test; DEPs of tumor (T) and adenoma (A) were tested using a two‐sample *t*‐test. *p*‐value < 0.05 and log(foldchange) > 1/log(foldchange) < −1 were used to determine differentially expressed host proteins between groups.

The 95% confidence intervals and *p* values for each microbial coefficient associated with a given host gene were obtained using the R package *“HDI”* and the results were plotted using the *ComplexHeatmap* package in R. The details are as described in the previous study [17].

#### CRC classification model based on intratumor microbial and protein characteristics

Based on differential intratumor microbial and protein signatures, a comprehensive analysis was performed to investigate potential microbial markers for CRC classification, which included cross‐validation model construction and independent validation. We used Gini importance ranking in the random forest algorithm to screen microbes and proteins with high contribution to the model. To construct predictive models, we tuned hyperparameters using the caret package. With the best combination of hyperparameters, we constructed a fivefold cross‐validation model to avoid overfitting issues. Furthermore, we used five additional datasets to perform independent validation analysis and test the robustness of features as CRC diagnostic markers. Like model construction in the discovery datasets, fivefold cross‐validation models were constructed with the identified best markers and evaluated with the average AUROC.

Proteomics research in CRC tumor tissue is indeed less extensive compared to genomics. Despite this, the central dogma of molecular biology, which outlines the flow of genetic information from DNA to RNA to proteins, suggests a direct relationship between mRNA and protein levels, a concept supported by studies showing significant correlations between RNA and protein levels. Thus, in testing the performance of our protein biomarkers in screening for CRC, we used transcriptome data.

Experimental statistical analyses were performed using GraphPad Prism v10.2.3 (GraphPad Software, CA, USA). Data are represented as mean ± SEM. Statistical significance between groups was determined using two‐way ANOVA, Kruskal‐Wallis test or unpaired two‐tailed Student's *t*‐test, as appropriate.

In the figures, significance levels were denoted as *p < 0.05, **p < 0.01, ***p < 0.001, and ****p < 0.0001.

## AUTHOR CONTRIBUTIONS

**Di Wu**: Validation; Visualization; writing—review and editing. **An‐Jun Wang**: Writing—original draft; visualization; formal analysis. **De‐Chao Bu**: Data curation; writing—review and editing; methodology. **Yan‐Yan Sun**: Writing—original draft; visualization; data curation. **Chen‐Hao Li**: Data curation; visualization. **Yue‐Mei Hong**: Validation. **Shan Zhang**: Visualization; formal analysis; data curation. **Shi‐Yang Chen**: Validation. **Jin‐An Zhou**: Validation. **Tian‐Yi Zhang**: Validation. **Min‐Hao Yu**: Resources. **Yong‐Jing Ma**: Visualization. **Xiu‐Li Wang**: Visualization. **Jia Xu**: Resources. **Wei He**: Resources. **Christopher Heeschen**: Writing—review and editing. **Jian‐Feng Chen**: Writing—review and editing. **Wen‐Jun Mao**: Writing—review and editing. **Hui Ding**: Resources. **Wen‐Juan Wu**: Resources; investigation. **Yi Zhao**: Methodology; visualization; data curation. **Hui Wang**: Project administration; writing—review and editing; investigation. **Ning‐Ning Liu**: Writing—review and editing; funding acquisition; project administration; supervision. All authors have read the final manuscript and approved it for publication.

## CONFLICT OF INTEREST STATEMENT

The authors declare no conflicts of interest.

## ETHICS STATEMENT

This study was conformed to the principles of the Helsinki Declaration. The animal experiments were performed in accordance with the Guide for the Care and Use of Laboratory Animals, which was approved by the Experimental Animal Ethical Committee at Shanghai Jiao Tong University School of Medicine (JUMC2024‐219‐A). Written informed consent was obtained from all participants before data and specimen collection, approved by the Ethics Committee of the Renji Hospital Affiliated to Shanghai Jiao Tong University School of Medicine (No. KY2021‐283‐B) and Shanghai Tenth People's Hospital (No. SHSY‐IEC‐4.1/20‐166/01).

## Supporting information


**Figure S1:** Inclusion and exclusion criteria of samples for the study cohort and overview of study design, related to Figure 1.
**Figure S2:** Changes in multi‐kingdom microbial composition in adenoma, para‐tumor, and tumor tissues.
**Figure S3:** Interaction network and functional prediction of multi‐kingdom tissue‐resident microbes in colorectal carcinogenesis.
**Figure S4:** Proteomic subtyping of adenoma, para‐tumoral and tumor tissues.
**Figure S5:** Alpha and beta diversity analysis in different proteomic subtyping.
**Figure S6:** Tissue‐resident microbe and proteins shared in adenoma, para‐tumoral and tumor tissues.
**Figure S7:** Co‐occurrence analyses of the mycotypes and bacteria, archaea, viruses, and immune cells using MMvec in early and advanced tumor tissues.
**Figure S8:** Performance of protein markers on external data sets.
**Figure S9:** Correlation between microbial biomarkers and functional pathways in different groups.

**Table S1:** Demographic characteristics of colorectal cancer (CRC) patients and colorectal adenoma (CRA) patients.
**Table S2:** List of microorganisms used for decontamination.
**Table S3:** Dataset for validation of annotated microorganisms.
**Table S4:** Sample sequence quality control statistics.
**Table S5:** The relative abundance of intratumor microorganisms in each sample at phylum level.
**Table S6:** The relative abundance of intratumor microorganisms in each sample at class level.
**Table S7:** The relative abundance of intratumor microorganisms in each sample at order level.
**Table S8:** The relative abundance of intratumor microorganisms in each sample at family level.
**Table S9:** The relative abundance of intratumor microorganisms in each sample at genus level.
**Table S10:** The relative abundance of intratumor microorganisms in each sample at species level.
**Table S11:** Differentially expressed microbes of A/T.
**Table S12:** Differentially expressed microbes of A/P.
**Table S13:** Differentially expressed microbes of T/P.
**Table S14:** Differentially expressed microbes of Early/Advanced.
**Table S15:** Categorization of microbial communities.
**Table S16:** Correlation between microbes and annotation pathways in T.
**Table S17:** Correlation between microbes and annotation pathways in P.
**Table S18:** Correlation between microbes and annotation pathways in Early.
**Table S19:** Correlation between microbes and annotation pathways in Advanced.
**Table S20:** Protein expression of each sample.
**Table S21:** Differentially expressed proteins of T/P.
**Table S22:** Differentially expressed proteins of T/A.
**Table S23:** Differentially expressed proteins P/A.
**Table S24:** Mycotype analysis.
**Table S25:** Host protein biomarkers for A/T and early/advanced stages of CRC.
**Table S26:** Correlation between microbial markers and annotation pathways in A.
**Table S27:** Correlation between microbial markers and annotation pathways in T.
**Table S28:** Correlation between microbial markers and annotation pathways in Early.
**Table S29:** Correlation between microbial markers and annotation pathways in Advanced.

## Data Availability

The metagenomic sequencing data of the study cohort are deposited in both the NCBI SRA under accession no. PRJNA956300 (https://dataview.ncbi.nlm.nih.gov/object/PRJNA956300?reviewer=gvsa4n7anpjtnd0tvi3jv7s8jt). The source data are not publicly available now due to restrictions in the informed consent and data protection regulations. Reasonable requests for data access can be directed to the principal investigator, Prof. Ning‐Ning Liu (liuningning@shsmu.edu.cn). If data access is approved, the data users are bound by a data access agreement. This includes responsibilities with respect to third‐party data sharing and maintaining participant privacy. Further responsibilities include a responsibility to acknowledge data sharing. Deidentified genomic and associated data from this study are available for ethically approved research. The mass spectrometry proteomics data have been presented in Table S20. All public transcriptomic datasets used to assess the model's generalizability can be found at GEO (https://www.ncbi.nlm.nih.gov/geo/), including: GSE164541, GSE41657, GSE22598, GSE37364, and GSE100179. This paper does not report original code. The data and scripts used are saved in GitHub https://github.com/FunGuyGroup/imt2025987. Supplementary materials (figures, tables, graphical abstract, slides, videos, Chinese translated version, and update materials) may be found in the online DOI or iMeta Science http://www.imeta.science/. The data that support the findings of this study are openly available in National Center for Biotechnology Information at https://www.ncbi.nlm.nih.gov/, reference number PRJNA956300.
