# Supplementary material for: The interplay between tissue‐resident microbiome and host proteins by integrated multi‐omics during progression of colorectal adenoma to carcinoma
Source: Imeta. 2025 Nov 4;4(6):e70090. doi: 10.1002/imt2.70090 (PMC12747540; doi:10.1002/imt2.70090)
Supplement: Supplementary file 1 — Figure S1: Inclusion and exclusion criteria of samples for the study cohort and overview of study design, related to Figure 1. Figure S2: Changes in multi‐kingdom microbial composition in adenoma, para‐tumor, and tumor tissues. Figure S3: Interaction network and functional prediction of multi‐kingdom tissue‐resident microbes in colorectal carcinogenesis. Figure S4: Proteomic subtyping of adenoma, para‐tumoral and tumor tissues. Figure S5: Alpha and beta diversity analysis in different proteomic subtyping. Figure S6: Tissue‐resident microbe and proteins shared in adenoma, para‐tumoral and tumor tissues. Figure S7: Co‐occurrence analyses of the mycotypes and bacteria, archaea, viruses, and immune cells using MMvec in early and advanced tumor tissues. Figure S8: Performance of protein markers on external data sets. Figure S9: Correlation between microbial biomarkers and functional pathways in different groups. [file IMT2-4-e70090-s001.docx]

**Supplementary information to**

# The interplay between tissue-resident microbiome and host proteins by integrated multi-omics during progression of colorectal adenoma to carcinoma

# Running title: Tissue-resident microbiome and host protein interaction in CRC

Di Wu^1#^, An-Jun Wang^1#^, De-Chao Bu^2,3#^, Yan-Yan Sun^1#^, Chen-Hao Li^2,3#^, Yue-Mei Hong^1^, Shan Zhang^2,3^, Shi-Yang Chen^4^, Jin-An Zhou^1^,Tian-Yi Zhang^1^, Min-Hao Yu^5^, Yong-Jing Ma^1^, Xiu-Li Wang^1^, Jia Xu^5^, Wei He^6^, Christopher Heeschen^1^, Jian-Feng Chen^4,7^, Wen-Jun Mao^8,9*^, Hui Ding^10,11*^, Wen-Juan Wu^12*^, Yi Zhao^2,3*^, Hui Wang^1*^, Ning-Ning Liu^1*^

1 State Key Laboratory of Systems Medicine for Cancer, Center for Single-Cell Omics, School of Public Health, Shanghai Jiao Tong University School of Medicine, Shanghai 200025, China.
2 Research Center for Ubiquitous Computing Systems, Institute of Computing Technology, Chinese Academy of Sciences, Beijing 100190, China.
3 University of Chinese Academy of Sciences, Beijing 100049, China.
4 Key Laboratory of Systems Health Science of Zhejiang Province, School of Life Science, Hangzhou Institute for Advanced Study, University of Chinese Academy of Sciences, Hangzhou 310024, China.
5 Department of Gastrointestinal Surgery, Renji Hospital, Shanghai Jiao Tong University School of Medicine, Shanghai 200127, China.
6 Department of Pathology, Renji Hospital, Shanghai Jiao Tong University School of Medicine, Shanghai 200127, China.
7 Department of Immunology, School of Basic Medical Sciences, Fudan University, Shanghai 200032, China.
8 Department of Thoracic Surgery, The Affiliated Wuxi People's Hospital of Nanjing Medical University, Wuxi People's Hospital, Wuxi Medical Center, Nanjing Medical University, Wuxi 214002, China.
9 Center of Clinical Research, The Affiliated Wuxi People's Hospital of Nanjing Medical University, Wuxi People's Hospital, Wuxi Medical Center, Nanjing Medical University, Wuxi 214002, China.
10 Department of Gastroenterology and Hepatology, Renji Hospital, Shanghai Jiao Tong University School of Medicine, Shanghai Jiao Tong University, Shanghai 200127, China.
11 Shanghai Institute of Digestive Disease, NHC Key Laboratory of Digestive Diseases, Shanghai 200127, China.
12 Department of Laboratory Medicine, Shanghai East Hospital, Tongji University School of Medicine, Shanghai, 200123, China.

^#^These authors contributed equally: Di Wu, An-Jun Wang, De-Chao Bu, Yan-Yan Sun, Chen-Hao Li.

* Correspondence:

liuningning@shsmu.edu.cn (Ning‐Ning Liu), huiwang@shsmu.edu.cn (Hui Wang), biozy@ict.ac.cn (Yi Zhao), wwj1210@126.com (Wen-Juan Wu), dr_daisy@163.com (Hui Ding), maowenjun1@njmu.edu.cn (Wen-Jun Mao).

**Supplementary figures**

**Figure S1 Inclusion and exclusion criteria of samples for the study cohort and overview of study design, related to Figure 1.** (A) Quality control steps of CRC and CRA cohort. (B) Overview of study design: colonic biopsy samples were collected from individuals in CRC and CRA cohort and for each sample, paired host proteomics data and tissue-resident microbiome metagenomics data were generated. The paired host proteomic and metagenomic data were integrated using a machine learning-based framework to characterize the tissue-resident microbiome-host protein associations in CRC (see Methods for details on the integration framework and mathematical notations). (C) Information and analysis for each patient. Each column represents a patient. On the left are CRC patients and on the right are CRA patients. (D) Flow chart of dealing with contaminations in metagenomics profiling of low microbial biomass samples. (E) The agarose gel electrophoresis result of PCR products of negative controls and clinical samples. Column A represents Trans2K Plus II DNA Marker. Columns B-I represent results under ITS2 primers. B-E columns represent clinical samples, F-G columns represent DNA extraction controls, Columns H-I represent environmental controls. Columns J-Q represent results under 16S primers. The order is the same as B-I. (F) Verification with reported microorganisms. Shared in the verification cohorts and the discovery cohort are green, specific in the verification cohorts are blue, and specific in the discovery cohort is orange. CRC, colorectal cancer; CRA, colorectal adenomas; PPI, proton pump inhibitors; BMI, body mass index. PCR, polymerase chain reaction; LASSO, least absolute shrinkage and selection operator; Sparce CCA, sparse canonical correlation analysis; IPA, ingenuity pathway analysis; TIDE, tumor immune dysfunction and exclusion; GSEA, gene set enrichment analysis; QC, quality control.

**Figure S2 Changes in multi-kingdom microbial composition in adenoma, para-tumor, and tumor tissues.** (A) Microbial composition of four kingdoms in A, T, and P in genus level. Color strength indicates the recurrence of microbes in the sample and Ratios represent the percentage of microbes. Only the abundant genus is shown in the bubble chart and the rare family are summed into others. (B) Microbial composition of four kingdoms in A, early, and advanced at genus level. Color strength indicates the recurrence of microbes in the sample and Ratios represent the percentage of microbes. Only the abundant species or genus are shown in the bubble chart and the rare family are summed into others. (C) Microbial composition of four kingdoms in A, early, and advanced at species level. Color strength indicates the recurrence of microbes in the sample and Ratios represent the percentage of microbes. Only the abundant species or genus are shown in the bubble chart and the rare family are summed into others. Freq, frequency.

**Figure S3 Interaction network and functional prediction of multi-kingdom tissue-resident microbes in colorectal carcinogenesis.** (A) The interaction network of all four kingdoms between each group at species level. Estimation of correlation using SparCC algorithm. The colours of nodes indicate species and the size of nodes indicate the abundance of species. Only significant absolute correlations above 0.3 are shown, which are considered as fair correlations. The red lines indicate negativeinteractions; the green lines indicate positive interactions. (B) The interaction network of all four kingdoms between *Ascomycota* and others. Estimation of correlation using SparCC algorithm. (C) The heatmap shows Spearman correlations between differential microbes and metabolic pathways in early and advanced for the top 50 most differential bacterial and top 10 fungal/archaeal/viral species-pathway pairs, ranked by absolute difference in correlation coefficients between groups.

**Figure S4 Proteomic subtyping of adenoma, para-tumoral and tumor tissues.** (A) The phylogenetic tree of the proteomics hierarchical clustering. All subjects were divided into four subgroups, named C1-C4. The bar above represents Cluster. Each row represents a patient sample. (B) Consensus clustering based on differentially expressed proteins. All subjects were divided into four subgroups, named C1-C4. The bars above the heatmap represent Stage, Group and Cluster, respectively. Each column represents a patient sample and rows indicate proteins.

**Figure S5 Alpha and beta diversity analysis in different proteomic subtyping.** (A) Microbiome diversity measured by Simpon index of different protein molecular subtypes. From left to right, alpha diversity of different molecular subtypes of C1-C4 are shown, with different colors representing different groups. *p* value (Kruskal-Wallis test) was calculated. Data are shown via the interquartile ranges (IQRs) with the median as a black horizontal line and the whiskers extending up to the most extreme points within 1.5× the IQR; outliers are represented as dots. NS, not significant. (B) Principal coordinate analysis (PCoA) based on Bray–Curtis distance, which shows that microbial composition was different between different protein molecular subtypes. *p* values calculated used two-sided permutation multivariate analysis of variance (PERMANOVA). (C) Spearman correlation analysis between the abundance of tumor tissue-enriched microbial species and Tumor immune dysfunction and exclusion (TIDE) scores in different subgroups. Microbial species shown are those significantly enriched in tumor (T) tissues compared to both adenoma (A) and para-tumor (P) tissues. Only significant correlations are annotated with asterisks. **p* < 0.05. (D) Heatmap showing the pattern of correlation between host proteins (rows) and tissue-resident microbial taxa (columns) identified by the LASSO model, shared in A, early, and advanced.

**Figure S6 Tissue-resident microbe and proteins shared in adenoma, para-tumoral and tumor tissues.** (A) Heatmap showing the pattern of correlation in A between host proteins (rows) and tissue-resident microbial taxa (columns) in A identified by the LASSO model, shared among the A, P, and T. (B) Heatmap showing the pattern of correlation in P between host proteins (rows) and tissue-resident microbial taxa (columns) in A identified by the LASSO model, shared among the A, P, and T. (C) Heatmap showing the pattern of correlation in T between host proteins (rows) and tissue-resident microbial taxa (columns) in A identified by the LASSO model, shared among the A, P, and T.

**Figure S7 Co-occurrence analyses of the mycotypes and bacteria, archaea, viruses, and immune cells using MMvec in early and advanced tumor tissues.** (A) Heatmap for inferring conditional probabilities in early. Co-occurrence analyses of fungal, bacteria, archaeal, viral species, and immune cell compositions using microbe–metabolite vectors (MMvec). Hierarchical clustering information identified three distinct clusters (‘‘mycotypes’’) associated with groups of fungal genera: F1, F2, and F3. (B) Heatmap for inferring conditional probabilities in advanced. Co-occurrence analyses of fungal, bacteria, archaeal, viral species, and immune cell compositions using MMvec. Hierarchical clustering information identified three distinct clusters (‘‘mycotypes’’) associated with groups of fungal genera: F1, F2, and F3.

**Figure S8 Performance of protein markers on external data sets.** (A) ROC curves as performance evaluation random-forest models and to distinguish adenomas from CRC patients. Different colors of the curve represent different data sets. (B) ROC curves as performance evaluation random-forest models and to distinguish early and advanced CRC patients. Different colors of the curve represent different data sets.

**Figure S9 Correlation between microbial biomarkers and functional pathways in different groups.** (A) Heatmap shows Spearman correlations between microbial markers and differentially metabolic pathways in A and T. (B) Heatmap shows Spearman correlations between microbial markers and differentially metabolic pathways in early and advanced tumor tissues.
